# Supplementary material for: Feasibility of data linkage in the PARAMEDIC trial: a cluster randomised trial of mechanical chest compression in out-of-hospital cardiac arrest
Source: BMJ Open. 2018 Jul 28;8(7):e021519. doi: 10.1136/bmjopen-2018-021519 (PMC6067361; doi:10.1136/bmjopen-2018-021519)
Supplement: Supplementary file 1 [file bmjopen-2018-021519supp001.pdf]

Supplementary materials:

Supplementary Table 1: Summary of retrieved information in each linked dataset.

HES Inpatient		All
CSK operation	No	606 (79.6%)
	Yes	155 (20.4%)
Destination of discharge	The usual place of residence, including no fixed abode	192 (24.9%)
	Temporary place of residence when usually resident elsewhere	4 (0.5%)
	Repatriation from high security psychiatric hospital (from 1999-2000)	0 (0.0%)
	Repatriation from high security psychiatric hospital (from 1999-2000)	0 (0.0%)
	Penal establishment - court (from 1999-2000)	0 (0.0%)
	Penal establishment - police station (from 1999-2000)	0 (0.0%)
	Penal establishment - court and police station excluded (from 1999-2000 to 2006-07)	0 (0.0%)
	High security psychiatric hospital, Scotland (from 1999-2000)	0 (0.0%)
	NHS other hospital provider - high security psychiatric accommodation	0 (0.0%)
	NHS other hospital provider - medium secure unit (from 1999-2000)	32 (4.2%)
	NHS other hospital provider - ward for maternity patients or neonates	0 (0.0%)
	NHS other hospital provider - ward for patients who are mentally ill or have learning disabilities	0 (0.0%)
	NHS run nursing home, residential care home or group home	3 (0.4%)
	Local authority Part 3 residential accommodation - where care is provided (from 1996-97)	0 (0.0%)
	Local authority foster care, but not in Part 3 residential accommodation - where care is provided (from 1996-97)	0 (0.0%)
	LA home or care (1989-90 to 1995-96)	0 (0.0%)
	Non-NHS run hospital - medium secure unit (from 2003-04)	1 (0.1%)
	Non-NHS (other than local authority) run residential care home (from 1996-97 to 2003-04) and care home (from 2003-04)	3 (0.4%)
	Non-NHS (other than local authority) run nursing home (from 1996-97 to 2003-04)	0 (0.0%)
	Non-NHS run hospital	0 (0.0%)
	Non-NHS (other than local authority) run hospice	2 (0.3%)
	Non-NHS institution (1989-90 to 1995-96)	0 (0.0%)
	Not applicable	7 (0.9%)
	Not known	1 (0.1%)
	Died	522 (67.7%)
Method of discharge	Discharged on clinical advice or with clinical consent	234 (30.4%)
	Self discharged, or discharged by a relative or advocate	5 (0.6%)

	Discharged by a mental health review tribunal, the Home Secretary or a court	0 (0.0%)
	Baby was still born	0 (0.0%)
	Not applicable: patient still in hospital	10 (1.3%)
	Not known: a validation error	0 (0.0%)
	Died	522 (67.7%)
IMD 2004 index	Missing	84 (10.9%)
	Least deprived 10%	64 (8.3%)
	Less deprived 10-20%	43 (5.6%)
	Less deprived 20-30%	47 (6.1%)
	Less deprived 30-40%	35 (4.5%)
	Less deprived 40-50%	50 (6.5%)
	More deprived 10-20%	80 (10.4%)
	More deprived 20-30%	84 (10.9%)
	More deprived 30-40%	58 (7.5%)
	More deprived 40-50%	61 (7.9%)
	Most deprived 10%	165 (21.4%)
Hospital length of stay*		7.7 (20.3)
**HES A&E**		**All**
A&E department type - A&E only	Emergency departments	1865 (96.8%)
	Consultant-led mono specialty accident and emergency service'	0 (0.0%)
	Other type of A&E. Excludes NHS walk-in centres	4 (0.2%)
	NHS walk-in centres	1 (0.1%)
	Not known	57 (3.0%)
A&E attendance disposal - A&E only	Admitted to hospital bed / became a lodged patient of the same health care provider	560 (29.1%)
	Discharged – follow-up treatment to be provided by general practitioner	5 (0.3%)
	Discharged – did not require any follow-up treatment	9 (0.5%)
	Referred to A&E clinic	13 (0.7%)
	Referred to fracture clinic	1 (0.1%)
	Referred to other outpatient clinic	1 (0.1%)
	Transferred to other healthcare provider	28 (1.5%)
	Referred to other healthcare professional	6 (0.3%)
	Left department before being treated	1 (0.1%)
	Left department having refused treatment	0 (0.0%)
	Other	5 (0.3%)
	Not known	2 (0.1%)
	Died in department	1296 (67.3%)
IMD 2004 index	Missing	7 (0.4%)
	Least deprived 10%	126 (6.5%)
	Less deprived 10-20%	95 (4.9%)
	Less deprived 20-30%	139 (7.2%)
	Less deprived 30-40%	120 (6.2%)

	Less deprived 40-50%	133 (6.9%)
	More deprived 10-20%	257 (13.3%)
	More deprived 20-30%	213 (11.1%)
	More deprived 30-40%	173 (9.0%)
	More deprived 40-50%	205 (10.6%)
	Most deprived 10%	459 (23.8%)
**HES Critical care**		**All**
Length of level 2 CC days		0.9 (1.7)
Length of level 3 CC days		4.4 (5.4)
Total length of CC days		6.6 (15.4)
**ICNARC CMP**		**All**
Length of level 0 ITU days		0 (0.3)
Length of level 1 ITU days		0.1 (0.6)
Length of level 2 ITU days		1.1 (4.0)
Length of level 3 ITU days		5.2 (9.4)
Total length of ITU days		6.5 (12.9)
Total length of ITU days in 30 days survived patients		10.5 (22.0)
Total length of ITU days in 30 days deceased patients		4.8 (4.5)
Days of alive and free of ITU stay in the first 28 days of cardiac arrest		6.9 (10.3)
Treatment withheld/withdrawn	Both withheld then withdrawn	23 (5.7%)
	Withheld	4 (1.0%)
	Withdrawn	133 (32.7%)
	Neither	247 (60.7%)
Organ donation	Heartbeating solid organ donor	13 (3.2%)
	No solid organs or tissues donated	199 (48.9%)
	Non-heartbeating solid organ donor	15 (3.7%)
	Tissue donor only	8 (2.0%)
**NICOR MINAP**		**All**
Admission Diagnosis	Definite myocardial infarction	126 (69.2%)
	Acute coronary syndrome	30 (16.5%)
	Chest pain cause	4 (2.2%)
	Other initial diagnosis	22 (12.1%)
Admission Ward	Missing	1 (0.5%)
	Cardiac care unit	94 (51.6%)
	Acute admissions unit	6 (3.3%)
	General medical ward	1 (0.5%)
	Intensive therapy unit	69 (37.9%)
	Other	9 (4.9%)
	Cardiac ward (non CCU)	1 (0.5%)
	Stepdown ward	0 (0.0%)
	Unknown	0 (0.0%)
	Died in A&E	1 (0.5%)

Initial Reperfusion Treatment	Missing	3 (2.2%)
	None	18 (13.1%)
	Thrombolytic treatment	0 (0.0%)
	pPCI in house	116 (84.7%)
	Referred for consideration for pPCI elsewhere	0 (0.0%)*
	Unknown	0 (0.0%)
Procedure performed	Missing	30(21.9%)
	No angiogram	2 (1.5%)
	Angiogram but no PCI	9 (6.6%)
	Angiogram and PCI	96 (70.1%)
	Unknown	0 (0.0%)
Coronary angiography	Missing	1(2.9%)
	Protocol driven investigation performed in this hospital	12(35.3%)
	Symptom driven investigation performed in this hospital	7 (20.6%)
	Protocol driven investigation performed at another hospital	0 (0.0%)
	Symptom driven investigation performed at another hospital	0 (0.0%)
	Planned after discharge	0 (0.0%)
	Not applicable	9 (6.6%)
	Patient refused	1 (2.9%)
	Not performed	5 (14.7%)
	Unknown	0 (0.0%)
Coronary intervention	Missing	6 (17.6%)
	Percutaneous coronary intervention	9 (26.5**%)**
	CABG	0 (0.0%)
	PCI planned after discharge	0 (0.0%)
	CABG planned after discharge	1(2.9%)
	Not applicable	5 (14.7%)
	Patient refused	1 (2.9%)
	Not performed or arranged	11 (32.4%)
	Unknown	0 (0.0%)
Assessment at non-interventional centre	Missing	5 (2.7%)
	No contact with a non interventional hospital	126 (69.2%)
	Patient remains in ambulance	0 (0.0%)
	A&E	30 (16.5%)
	Acute assessment unit	2 (1.1%)
	CCU / cardiac facility	1 (0.5%)
	Self referral	0 (0.0%)
	Already in hospital	2 (1.1%)
	Other	0 (0.0%)
	Unknown	16 (8.8%)
Assessment at interventional centre	Missing	59 (32.4%)
	Assessed in A&E	68 (37.4%)
	Acute assessment unit	1 (0.5%)
	CCU / cardiac facility	26 (14.3%)

	Catheter laboratory	28 (15.4%)
	Already in hospital	0 (0.0%)
	Unknown	0 (0.0%)
**NICOR PCI**		**All**
Cardiopulmonary resuscitation (CPR)	Missing	29 (28.7%)
	No	72 (71.3%)
	Yes	0 (0.0%)
Coronary artery bypass grafting (CABG)	Missing	0 (0.0%)
	No	101 (100%)
	Yes	0 (0.0%)
Ventilation	Missing	101 (100%)*
	No	0 (0.0%)
	Yes	0 (0.0%)
Hypothermia	Missing	101 (100%)*
	No	0 (0.0%)
	Yes	0 (0.0%)
Percutaneous coronary intervention (PCI)	Missing	101 (100%)
	No	0 (0.0%)
	Yes	0 (0.0%)
	No	0 (0.0%)
	Yes	0 (0.0%)

Note: *: Variables were added to the dataset after the linkage was performed.

Supplementary Table 2: Summary of demographic and cardiac arrest event characteristics in trial patients with and without matched HES inpatient, Critical care and A&E data

		HES matched N=2079	HES unmatched N=319	p value
Age (year)		73.6 (21.9)	71.8 (24.4)	0.151
Sex	Male	1315 (63.3%)	215 (67.4%)	0.151
	Female	764 (36.8%)	104 (32.6%)	
Initial aetiology	Presumed cardiac	1786 (85.9%)	272 (85.3%)	0.864
	Respiratory	146 (7.0%)	27 (8.5%)	
	Submersion	8 (0.4%)	1 (0.3%)	
	Other	74 (3.6%)	9 (2.8%)	
	Unknown	65 (3.1%)	10 (3.1%)	
Initial rhythm	VF	624 (30.0%)	99 (31.0%)	0.468
	VT	20 (1.0%)	1 (0.3%)	
	PEA	613 (29.5%)	83 (26.0%)	
	Asystole	727 (35.0%)	118 (37.0%)	
	Unknown	95 (4.6%)	18 (5.6%)	
Location	Home	1610 (77.4%)	206 (64.6%)	<0.001
	Public place	333 (16.0%)	89 (27.9%)	
	Other	136 (6.5%)	24 (7.5%)	
Witness	Not witnessed	508 (24.5%)	60 (18.8%)	0.027
	By bystander	961 (46.3%)	170 (53.3%)	
	By EMS	413 (19.9%)	60 (18.8%)	
	By Non-EMS healthcare	64 (3.1%)	4 (1.3%)	
	Unknown	132 (6.4%)	25 (7.8%)	
Cardiopulmonary resuscitation (CPR) by Bystander	No	1104 (53.1%)	156 (48.9%)	0.376
	Yes	862 (41.5%)	144 (45.1%)	
	Unknown	113 (5.4%)	19 (6.0%)	
Response time (minute)†		6.1 (4.3)	7.2 (5.1)	<.0001
Survival at 30days	Alive	224 (10.8%)	38 (11.9%)	0.544
	Deceased	1855 (89.2%)	281 (88.1%)	
ROSC at hospital transfer	ROSC	790 (38.0%)	125 (39.2%)	<0.001
	CPR in progress	1204 (57.9%)	135 (42.3%)	
	Unknown	85 (4.1%)	59 (18.5%)	
EQ5d at 3 months		70.0 (20.0)	75.0 (20.0)	0.970
EQ5d at 12 months		80.0 (30.0)	72.5 (26.0)	0.368
SF12 mental health at 3 months		50.4 (16.8)	45.4 (23.0)	0.690
SF12 physical health at 3 months		40.8 (14.2)	44.1 (15.0)	0.533
SF12 mental health at 12 months		51.0 (14.5)	44.1 (10.5)	0.118
SF12 physical health at 12 months		45.2 (17.4)	38.4 (13.9)	0.059
HADS Anxiety at 12 months		5.0 (7.0)	6.5 (5.0)	0.112

HADS depression at 12 months	4.0 (5.0)	6.0 (4.5)	0.163
MMSE at 12 months	29.0 (3.0)	29.0 (2.0)	0.648
PTSD at 12 months	27.0 (17.0)	33.0 (19.0)	0.280

Note: Continuous variables were shown as median (Interquartile range) and categorical variables were shown as n (percentage). †: response time was from 999 call to EMS arrival at scene.

Supplementary Table 3: Summary of demographic and cardiac arrest event characteristics in patients with matched HES Critical care only, CMP only and both.

		HES Critical Care only	ICNARC CMP only	Critical care & CMP	p value
Age (year)		68.3 (24.6)	64.8 (28.2)	65.5 (21.2)	0.684
Sex	Male	36 (70.6%)	66 (63.5%)	208 (68.7%)	0.556
	Female	15 (29.4%)	38 (36.5%)	95 (31.4%)	
Aetiology	Presumed cardiac	45 (88.2%)	84 (80.8%)	247 (81.5%)	0.554
	Respiratory	1 (2.0%)	12 (11.5%)	25 (8.3%)	
	Submersion	0 (0.0%)	1 (1.0%)	1 (0.3%)	
	Other	3 (5.9%)	6 (5.8%)	20 (6.6%)	
	Unknown	2 (3.9%)	1 (1.0%)	10 (3.3%)	
Location	Home	37 (72.6%)	68 (65.4%)	209 (69.0%)	0.516
	Public place	14 (27.5%)	30 (28.9%)	79 (26.1%)	
	Other	0 (0.0%)	6 (5.8%)	15 (5.0%)	
Witness	No	11 (21.6%)	26 (25%)	69 (22.8%)	0.981
	By bystander	29 (56.9%)	59 (56.7%)	174 (57.4%)	
	By EMS	7 (13.7%)	13 (12.5%)	32 (10.6%)	
	By Non-EMS healthcare	1 (2.0%)	2 (1.9%)	9 (3.0%)	
	Unknown	3 (5.9%)	4 (3.9%)	19 (6.3%)	
Cardiopulmonary resuscitation	No	24 (47.1%)	45 (43.3%)	136 (44.9%)	0.802
	Yes	24 (47.1%)	52 (50.0%)	155 (51.2%)	
	Unknown	3 (5.9%)	7 (6.7%)	12 (4.0%)	
Rhythm	VF	26 (51%)	44 (42.3%)	153 (50.5%)	0.437
	VT	2 (3.9%)	1 (1.0%)	4 (1.3%)	
	PEA	10 (19.6%)	25 (24.0%)	55 (18.2%)	
	Asystole	11 (21.6%)	26 (25.0%)	80 (26.4%)	
	Unknown	2 (3.9%)	8 (7.7%)	11 (3.6%)	
Response time (minute)†		6.6 (4.4)	6.7 (4.7)	6.0 (3.9)	0.034
Survival at 30days	Alive	19 (37.3%)	31 (29.8%)	93 (30.7%)	0.606
	Deceased	32 (62.8%)	73 (70.2%)	210 (69.3%)	
ROSC at hospital transfer	ROSC	42 (82.4%)	79 (76%)	241 (79.5%)	0.294
	CPR in progress	7 (13.7%)	14 (13.5%)	47 (15.5%)	
	Unknown	2 (3.9%)	11 (10.6%)	15 (5.0%)	
EQ5d at 3 months		67.5 (24.0)	75.5 (16.0)	70.0 (25.0)	0.838
EQ5d at 12 months		80.0 (14.0)	74.0 (20.0)	80.0 (25.0)	0.298
SF12 mental health at 3 months		48.4 (11)	48.3 (19.3)	49.3 (16.5)	0.794
SF12 physical health at 3 months		38.9 (13.2)	39.2 (13)	41.1 (14.2)	0.836
SF12 mental health at 12 months		51.6 (8)	43 (14.3)	47.8 (15.2)	0.543
SF12 physical health at 12 months		42.9 (21)	38.8 (14.6)	46.7 (15.6)	0.235

HADS Anxiety at 12 months	5.0 (5.0)	9.5 (6.5)	6.0 (6.0)	0.078
HADS depression at 12 months	4.0 (3.0)	6.0 (5.5)	5.0 (8.0)	0.368
MMSE at 12 months	28.0 (3.0)	29.0 (3.0)	29.0 (3.0)	0.717
PTSD at 12 months	30.0 (8.5)	40.0 (23.0)	29.0 (15.0)	0.631

Note: Continuous variables were shown as median (Interquartile range) and categorical variables were shown as frequency (percentage). †: response time is from 999 call to EMS arrival at scene.
