# Supplementary material for: Feasibility of data linkage in the PARAMEDIC trial: a cluster randomised trial of mechanical chest compression in out-of-hospital cardiac arrest
Source: BMJ Open. 2018 Jul 28;8(7):e021519. doi: 10.1136/bmjopen-2018-021519 (PMC6067361; doi:10.1136/bmjopen-2018-021519)
Supplement: Author's manuscript [file bmjopen-2018-021519.draft_revisions.pdf]

BMJ Open

BMJ Open is committed to open peer review. As part of this commitment we make the peer review history of every article we publish publicly available.

When an article is published we post the peer reviewers' comments and the authors' responses online. We also post the versions of the paper that were used during peer review. These are the versions that the peer review comments apply to.

The versions of the paper that follow are the versions that were submitted during the peer review process. They are not the versions of record or the final published versions. They should not be cited or distributed as the published version of this manuscript.

BMJ Open is an open access journal and the full, final, typeset and author-corrected version of record of the manuscript is available on our site with no access controls, subscription charges or pay-per-view fees (http://bmjopen.bmj.com).

If you have any questions on BMJ Open's open peer review process please email info.bmjopen@bmj.com

BMJ Open

Feasibility of data linkage in the PARAMEDIC Trial: a cluster randomised trial of mechanical chest compression in out of hospital cardiac arrest

Journal:	*BMJ Open*
Manuscript ID	bmjopen-2018-021519
Article Type:	Research
Date Submitted by the Author:	11-Jan-2018

Complete List of Authors:	Ji, Chen; University of Warwick, Warwick Clinical Trials Unit<br>Quinn, Tom; St George's Univesity of London & Kingston University, Faculty of Health, Social Care & Education<br>Gavalova, Lucia; St George's Univesity of London & Kingston University, Faculty of Health, Social Care & Education<br>Lall, Ranjit; University of Warwick, Warwick Clinical Trials Unit<br>Scomparin, Charlotte; University of Warwick, Warwick Clinical Trials Unit<br>Horton, Jessica; University of Warwick, Warwick Clinical Trials Unit<br>Deakin, Charles; University Hospital Southampton NHS Foundation Trust, NIHR Southampton Respiratory Biomedical Research Unit<br>Pocock, Helen; South Central Ambulance Service NHS Foundation Trust<br>Smyth, Michael; University of Warwick, Warwick Clinical Trials Unit<br>Rees, Nigel; Welsh Ambulance Service NHS Trust<br>Brace-McDonnell, Samantha; University of Warwick, Warwick Clinical Trials Unit<br>Gates, Simon; University of Warwick, Warwick Clinical Trials Unit<br>Perkins, Gavin; University of Warwick, Warwick Clinical Trials Unit; Heart of England NHS Foundation Trust

Keywords:	Adult cardiology < CARDIOLOGY, Clinical trials < THERAPEUTICS, Data linkage, Cardiac arrest

SCHOLARONE™
Manuscripts

Feasibility of data linkage in the PARAMEDIC Trial: a cluster randomised trial of mechanical chest compression in out of hospital cardiac arrest.

Chen Ji[1] (C.Ji.3@warwick.ac.uk)

Tom Quinn[2] (T.Quinn@sgul.kingston.ac.uk)

Lucia Gavalova[2] (L.Gavalova@sgul.kingston.ac.uk)

Ranjit Lall[1] (R.Lall@warwick.ac.uk)

Charlotte Scomparin[1] (C.Scomparin@warwick.ac.uk)

Jessica Horton[1] (jessicahorton79@hotmail.com)

Charles D Deakin[3,4] (charlesdeakin@doctors.org.uk)

Helen Pocock[4] (Helen.Pocock1@nhs.net)

Michael A Smyth[1] (M.A.Smyth@warwick.ac.uk)

Nigel Rees[5] (Nigel.Rees5@wales.nhs.uk)

Samantha J Brace-McDonnell[1,6] (S.Brace-Mcdonnell@warwick.ac.uk)

Simon Gates[1] (Simon.Gates@warwick.ac.uk)

Gavin D Perkins[1,6] (G.D.Perkins@warwick.ac.uk)

1. Warwick Clinical Trials Unit, University of Warwick, Coventry, UK

2. Faculty of Health, Social Care and Education, Kingston University and St George's, University of London, London, UK

3. NIHR Southampton Respiratory Biomedical Research Unit, University Hospital Southampton NHS Foundation Trust, Southampton, Hampshire, UK

4. South Central Ambulance Service NHS Foundation Trust, Otterbourne, UK

5. Welsh Ambulance Service NHS Trust, Cardiff, UK

6. Heart of England NHS Foundation Trust, Birmingham, UK

Correspondence to

Professor Gavin Perkins; University of Warwick, Tel: +44 (0)24761 50925; G.D.Perkins@warwick.ac.uk

For peer review only - http://bmjopen.bmj.com/site/about/guidelines.xhtml

1 Word count

2 Abstract: 287

3 Main body: 3525 (excluding Tables/Figures/titles/notes/legends)

For peer review only

For peer review only - http://bmjopen.bmj.com/site/about/guidelines.xhtml

Abstract

Objectives: There is considerable interest in reducing the cost of clinical trials. Linkage of trial data to administrative datasets and disease-specific registries may improve trial efficiency, but has not been reported in resuscitation trials conducted in the UK. To assess the feasibility of utilising national administrative and clinical datasets to follow up patients transported to hospital following attempted resuscitation in a cluster randomised trial of a mechanical chest compression device in out of hospital cardiac arrest (OHCA).

Methods: Hospital data on trial participants were requested from Hospital Episode Statistics (HES); the Intensive Care National Audit and Research Centre (ICNARC); and Myocardial Ischaemia National Audit Project (MINAP) and National Audit of Percutaneous Coronary Intervention (NAPCI), using unique patient identifiers. Linked data were received between June 2014 and June 2015.

Results: Of 4471 patients randomised in the PARAMEDIC trial, 2398 (53.6%) were not known to be deceased at emergency department arrival and were eligible for linkage. We achieved an overall match rate of 86.7% in the combined HES A&E, inpatient and Critical care dataset, with variable match rates (4.2-80.4%) in individual datasets. No strong evidence of substantial bias was found in patient demographics, cardiac arrest related characteristics and major outcomes between HES matched and unmatched groups, in the linkage apart from location, response time and ROSC at handover.

Conclusions: This study shows that it is feasible to track patients from the pre-hospital setting through to hospital admission using routinely available administrative datasets with a moderate to high degree of success. This approach has the potential to complement the trial data with the demographic and clinical management information about the studied cohort, as well as to improve the efficiency and reduce the costs of follow-up in cardiac arrest trials.

ISRCTN0833942.

For peer review only - http://bmjopen.bmj.com/site/about/guidelines.xhtml

Strengths and limitations of this study

- First study evaluating the supplement of routinely collected administrative data in a cardiac arrest trial in the UK.
- Data linkage was made to different UK national registries.
- The matching reliability was suboptimal due to relaxed matching criteria, matching method and possible data quality issues.
- Routine data were not fully available for all trial patients transported to hospital.
- The findings of our study are not generalisable to facilitate trial recruitment since it was considered unrealistic in the clinical context of cardiac arrest.

For peer review only

For peer review only - http://bmjopen.bmj.com/site/about/guidelines.xhtml

Background

Well conducted and reported randomised controlled trials (RCTs) are considered the gold standard in evaluation of new or established clinical interventions. In cardiac arrest resuscitation science, only a small minority (1%) of contemporary international guideline recommendations are based on the highest level of evidence from more than one RCT, meta-analysis of high quality RCTs, or RCTs corroborated by high quality registry studies.[1] High quality trials to address outcomes of interest to patients following cardiac arrest (e.g. long term survival, neurocognitive status and disability)[2] are complex, labour intensive and expensive to perform. Many studies in cardiac arrest are therefore too small or inadequately conducted (with a predominance of observational studies which are prone to bias) to provide reliable estimates of treatment effect or harm to patients. Consequently, for the majority of resuscitation interventions, there is a paucity of high quality evidence. Funders (typically government agencies) have called for proposals for low-cost, more efficient trials.[3]

Traditional trial methods of patient tracking and data access in individual hospitals is challenging with limited resources. Cardiovascular medicine has attempted to improve the efficiency of the trial design by pioneering the concept of registry-based randomised trials, using clinical quality registries and administrative datasets. In the Thrombus Aspiration during ST-Segment Elevation Myocardial Infarction (TASTE) trial, undertaken in Sweden, both patient enrolment and follow up were conducted using the Swedish Web System for Enhancement and Development of Evidence-based Care in Heart Disease Evaluated According to Recommended Therapies (SWEDEHEART) registry.[4] On publication, this registry-based trial was hailed as the '*next disruptive technology*' in clinical research, and as a new clinical trial paradigm.[5 6] Subsequent registry-based trials have been reported in a comparison of radial versus femoral access in women undergoing percutaneous coronary intervention in the United States,[7] and of supplemental oxygen versus ambient air in patients with suspected acute myocardial infarction in Sweden.[8]

To our knowledge, however, there are no reports of registry-based randomised trials in resuscitation science. However, should accessing registry data to ascertain outcomes in a prehospital cardiac arrest trial (e.g. length of stay/patient pathways/survival status) to be feasible, this could be one way of significantly improving efficiency and reducing costs of conducting high quality randomised trials in resuscitation.

In the PARAMEDIC trial, the in-hospital data collection process was complex, expensive and labour intensive, with research paramedics visiting multiple hospitals across large geographical areas to extract data from hospital records. Patients transported to hospital following resuscitation from cardiac arrest follow multiple clinical pathways depending on their clinical status and treatments. As

For peer review only - http://bmjopen.bmj.com/site/about/guidelines.xhtml

For peer review only

hospital data are routinely collected and managed by national registries, utilising these registries could save resources and time in the in-hospital data collection and potentially reduce the burden on patients and relatives in the sensitive period following cardiac arrest.

This paper reports our assessment of the feasibility of linking data collected for the purposes of patient follow up in a pragmatic, cluster randomised controlled trial of a mechanical chest compression device undertaken in the United Kingdom (UK) prehospital setting, with large national administrative and specialist registries.

Methods

The PARAMEDIC trial examined the effectiveness of LUCAS-2, a mechanical chest compression device, in 4471 patients with out of hospital cardiac arrest (OHCA). The study was a cluster randomised trial whereby emergency medical service (EMS) vehicles were randomised to carry the LUCAS-2 device (intervention) or not (control). Full details of the trial protocol have been published previously.[9] In summary, adults with OHCA where resuscitation was attempted by EMS personnel and attended by a trial vehicle were included. Patients with traumatic cardiac arrest or suspected to be pregnant were excluded. Trial recruitment ran from April 15, 2010 to June 10, 2013. We have previously reported primary outcome (30-day survival),[10] secondary outcomes,[11] an economic analysis[12] and characteristics of patients who were not resuscitated.[13]

Data sources

The PARAMEDIC trial utilised four sources of data that were linked to the trial dataset: UK National Health Service (NHS) Hospital Episodes Statistics (HES), Myocardial Ischaemia National Audit Project (MINAP),[14] National Audit of Percutaneous Coronary Interventions (NAPCI),[15] and Case Mix Programme (CMP)[16] to obtain data on hospital stay and treatment or procedures that trial patients received in hospital.

We used the MINAP, NAPCI and CMP data for the health economic analysis[12] and long-term post admission outcomes[11] and to validate the hospital length of stay or stay in the intensive care (secondary outcomes for the efficacy part of the trial), and also to gain insight into the specifics of the treatment or procedures that trial patients received during their hospital stay. Characteristics of the registries are summarised in Table 1.

Table 1: Characteristics of registries, participation and case ascertainment.

Registry/Dataset	Source	Description	Participation and case ascertainment* during the trial period

For peer review only

Paramedic trial	Warwick Clinical Trials Unit	Trial patient cohort that survived admission to a hospital	n/a
Hospital Episode Statistics (HES)	NHS Digital (NHSD)	Collection of information on all NHS hospital inpatients, Accident and Emergency (A&E), critical care and outpatients which enables health care providers to be paid according to their levels of activity.	All hospitals<br>Case ascertainment 100%
Case Mix Programme (CMP)	Intensive Care National Audit and Research Centre (ICNARC)	Audit of patient outcomes from all adult, general critical care units in England, Wales and Northern Ireland. Other specialist units, including neurosciences, cardiac and high dependency units, also participate	Over 90% of critical care units<br><br>Case ascertainment not reported
Myocardial Ischaemia National Audit Project (MINAP)	National Institute for Cardiovascular Outcomes Research (NICOR)	National audit of patients with acute coronary syndrome admitted to all hospitals in England, Wales and Northern Ireland. Data are collected prospectively at each hospital by secure electronic system, electronically encrypted and transferred online to a central database	All hospitals<br><br>Case ascertainment not reported
NAPCI (National Audit of Percutaneous Coronary Interventions)	National Institute for Cardiovascular Outcomes Research (NICOR)	National audit of all PCI procedures from NHS and non-NHS hospitals in the United Kingdom.	All hospitals<br><br>Case ascertainment 97%

Note: *: Case ascertainment – Rate (e.g. %) of eligible cases included in a registry/database.

Patient population

Patients (denominator) for this linkage study were patients from the PARAMEDIC trial who were transported to hospital by EMS and not known to be deceased (i.e. documented as alive or unknown status) on arrival at the emergency department (ED).

Since NHS Digital, responsible for HES, only provides annual data up to 1st April each year, no data on trial patients recruited on or after 1st April 2013 had any HES data returned for this data request. We therefore limited our analysis of the linked registry data to patients recruited to the PARAMEDIC trial between April 2010 and March 2013.

Study approvals

The PARAMEDIC trial was approved by the Coventry Research Ethics Committee (reference 09/H1210/69) and sponsored by the University of Warwick, UK. The study was conducted in accordance with the principles of Good Clinical Practice and the Mental Capacity Act (2005). Specific approval for access to personal data without consent and the data linkage reported in this paper was obtained from the Confidentiality Advisory Group, part of the Health Research Authority (reference:

For peer review only

For peer review only - http://bmjopen.bmj.com/site/about/guidelines.xhtml

ECC 2-02 (c)/2011). At the time of the study this activity was undertaken by the National Information Governance Board for Health and Social Care Ethics and Confidentiality Committee.

Data linkage procedure

Data access applications were submitted to national administrative and disease registries between 2012-2014 to request patient case mix and clinical variables (Supplementary Table 1). The following patient identifiers were sent to the NHSD, ICNARC and NICOR to identify their clinical records: trial number, cardiac arrest date, ambulance service case number, 999 call time, hospital name, hospital arrival time, hospital handover time, patient name, NHS number, home address and postcode. The trial data were linked to the two NICOR datasets (MINAP and NAPCI) on two separate occasions by a different member of NICOR staff, which reassuringly generated the same results. Extracted anonymous data were encrypted and sent back to the trial team between June 2014 and June 2015.

Linked data may contain multiple, non-event related hospital records within the requested linkage period. We firstly used patient cardiac arrest (trial event) date to identify the records with exactly matched admission/visit date in the respective data sources. However, event and admission dates could be different due to potential data definition discrepancies. For instance, a trial event could occur before midnight and the patient was admitted to hospital after midnight. Therefore, we relaxed the date match criterion to a 5-day range (date of cardiac arrest with +/- 2 days). A matched record was redefined as if the admission/visit date falls in the range. We considered the range would be sufficiently large to mitigate against any date discrepancies in different sources and also be reasonably small to reduce the chance of mismatch in the case of early re-admission. Where multiple records could be matched to a single trial event in the same routine dataset, separate rules were used to extract the retrieved information: 1) where a patient had multiple episodes in HES, only the one with recorded death or discharge date was retained. If a patient had not been discharged from hospital, the episode with latest ward admission date was used. 2) Where multiple admissions to ICU were recorded in CMP, only the first ICU admission was linked to a trial event. 3) Since the MINAP dataset provided to us by NICOR only contained year and month of admission, only the earliest admission was used. 4) Only the first procedure was included for the linkage to the NAPCI registry data, since patients can have more than one interventional procedure (and thus another record) during the index admission.

Data linkage rate

For HES data, we developed the linkage and match rate for linked and matched (or correctly linked) cases as follows:

For peer review only - http://bmjopen.bmj.com/site/about/guidelines.xhtml

HES linkage rate = $\frac{\textbf{N of patients with linked HES inpatient, Critical care or A\&E data}}{\textbf{N of patients not known to be deceased at ED}}$

HES match rate = $\frac{\textbf{N of patients with matched (correctly linked) HES inpatient, Critical care or A\&E data}}{\textbf{N of patients not known to be deceased at ED}}$

Similar equations were used to determine the rates for each of the datasets i.e. MINAP, NAPCI and CMP. As we were not able to confirm which patient should actually be collected in these datasets, we employed same denominator used in the above equations.

Data linkage quality

Match rank is an indicator used in HES to show the confidence of match: 1 suggests the best match and 8 the worst. Level 1-3 appear to be of high quality as cases are matched based on a combination of unique NHS number and data of date of birth, sex and home postcode. The quality of linkage in matched HES was therefore summarised on the basis of percentage of level 1-3.

Data representativeness

Data representativeness was assessed in two comparisons. The first comparison intended to assess whether the patients with correctly linked (i.e. matched) HES data could be representative of the trial population. It was carried out in patients with and without matched HES inpatient, Critical Care or A&E data (comparison 1). The second comparison intended to assess the difference between two critical care data sources. We were not able to compare data from these two sources directly as some patient care data were collected in both databases. Hence, we split the patients by their linked data sources and made the comparison between patients with HES Critical Care only, with CMP data only and with both HES Critical Care and CMP data (comparison 2).

For both comparisons, we compared patient and event characteristics between the datasets. Continuous variables were compared using Mann-Whitney test in comparison 1 and Kruskal-Wallis test in comparison 2. Categorical variables were compared using Chi-square test. A two-sided p value <0.05 was considered statistically significant. All analyses were conducted in SAS v9.3 (Cary, NC, USA).

Data security and destruction

We followed the Warwick Clinical Trials Unit Standard Operating Procedures (SOPs) for data storage, transfer, and data sharing. The data were retained and destroyed in accordance with relevant regulations and the University of Warwick's Data Sharing Agreements.

Results

For peer review only - http://bmjopen.bmj.com/site/about/guidelines.xhtml

In the PARAMEDIC trial, 2695 patients were transported to hospital and not known to be deceased at ED. Of these 2398 (89.0%) were recruited between April 2010 and March 2013 and were therefore included in this study (referred to as “linkage patients”). The data requests to NHSD, ICNARC and NICOR retrieved different numbers of patient clinical records.

Summary of the linkage

The flow chart of the linkage to HES is shown in Figure 1. The linkage patients were grouped into ICU admitted (patients with matched HES Critical care data) and not admitted (patients with other matched HES data). Meanwhile, patients with matched CMP data were also summarised in the flowchart. This presented a comparison between CMP and HES Critical Care. 303 patients were matched in both CMP and HES Critical Care. Overall, the linkage to HES data achieved a match rate of 86.7% (2079 of 2398) with allowed variation in dates (date of cardiac arrest with +/- 2 days), slightly improved from the use of exact date match approach (84.1%).

Linkage quality was high in matched cases: level 1-3 accounted for 97.9%. In unmatched cases, 91.5% (292 of 319) had no linked HES data and the rest, while linked with non-trial even related data, had a good match rank (<=3).

The summary of linkage and match rate in each dataset are shown in Table 2. All datasets contained multiple linked records, indicating some patients had been linked to multiple admissions with possible multiple episodes. Among the 2398 linkage patients, individual match rate varied depending on the hospitalisation stage and received treatments. HES A&E had the highest individual match rate (80.4%). In the patients admitted to ICU, CMP provided 53 more matched patients with a lower proportion of unmatched data in linked patients compared to HES Critical Care.

Figure 1: Linkage match rate and flowchart of patients retrieving HES or CMP data.

Table 2: Summary of linked PARAMEDIC trial patients to the respective registry databases

Data source	Dataset	Number of linked records	Number of linked patients (linkage rate)*	Number of matched patients (match rate)*
NHSD	HES Inpatient	12875	1617 (67.4%)	771 (32.2%)
	HES Critical care	545	433 (18.1%)	354 (14.8%)
	HES A&E	6434	2186 (91.2%)	1927 (80.4%)

For peer review only - http://bmjopen.bmj.com/site/about/guidelines.xhtml

	Overall	19854	2277 (95.0%)	2079 (86.7%)
ICNARC	CMP	435	410 (17.1%)	407 (17.0%)
NCAP	MINAP	244	218 (9.1%)	182 (7.6%)
	PCI	153	128 (5.3%)	101 (4.2%)

Note: *: Percentage is calculated using the number in the column divided by 2398 linkage patients.

A summary of retrieved information for each linked dataset as well as the degree of data missingness for each field is available in the online supplementary materials. In Supplementary Table 2, the trial patients that had not been matched to the HES records were similar to those that with matched records in age (mean age 71.8 and 73.6 respectively), male (67.4% and 63.3% respectively were male). They were also similar between groups in initial cardiac arrest aetiology where most were of cardiac origin (85.3% and 85.9% respectively) and in initial rhythm (shockable rhythm 31.0% and 31.3%). Patients with unmatched data were more likely to have had a cardiac arrest in a public place (27.9%) compared with of those with matched records (16%), witnessed by bystander (53.3% versus 46.3%) and had longer EMS response time (7.2 versus 6.1 minutes). Supplementary Table 3 illustrates the comparison of demographic and event characteristics of patients with matched HES Critical Care and CMP data. Characteristics were similar in all three groups, except for a significant difference in the EMS response time.

Discussion

This study aimed to demonstrate the feasibility of collecting trial outcome data during patient follow up in a prehospital cardiac arrest trial via linkage to national registries. We achieved an overall match rate of 86.7% in 2398 patients using HES data. The data linkage provided important administrative and additional clinical data that allowed extended analyses of the intervention effect and provided more details of patient journey in the trial. We also evaluated the representativeness of retrieved HES and CMP data by comparing patient and trial event characteristics. No substantial difference was found in patients with and without matched HES inpatient, Critical Care or A&E data, as well as in patients with matched HES Critical Care only, CMP only and both datasets.

This was the first study evaluating the supplement of routinely collected administrative data in a cardiac arrest trial in the UK. Our match rate was in line with observational studies linking EMS data to hospital records,[17-20] and data validation studies.[21] Our experience suggests it is feasible to obtain relevant data from administrative databases in a cardiac arrest trial. In addition to the high match rate reported in this paper, the matched data are deemed to be sufficiently representative of the trial population. The comparison between patients with and without matched HES showed low level

For peer review only

For peer review only - http://bmjopen.bmj.com/site/about/guidelines.xhtml

of imbalance of event characteristics. Overall the results suggested no substantial bias. We have found similar results in the matched ICU data.

The unmatched cases were likely to be associated with missing or inaccurate data. Data quality could be at increased risk due to the challenging circumstances of cardiac arrest and complexity of patient handling following hospital arrival. In addition, routine data in the chosen registries are not systematically adjudicated. Lack of clinical engagement may compromise the case ascertainment and data quality,[22] leading to suboptimal linkage. NHS Digital employs deterministic and probabilistic methods in the data linkage. The latter calculates probability weight based on combinations of linkage variables and determines linkage based on a cut-off threshold. Although this method largely improves the linkage, it could incorrectly link record pairs and miss valid ones, undermining the reliability of linkage.

Linkage to individual routine datasets resulted in variable match rates. HES A&E generated the highest rate of 80.4% as most patients were taken by EMS to ED for assessment before being admitted to specialist hospital units. Other rates reflected the proportion of specific groups of patients in the linkage. The CMP, MINAP and NAPCI registries are focused on selected patients with a specific diagnosis and/or requiring specialist care, reflected in strict inclusion and exclusion criteria; for example MINAP comprises data on patients with suspected and/or confirmed acute coronary syndrome, NAPCI on interventional cardiology whilst CMP registry collects data on patients admitted to critical care/intensive care units within any given hospital. In this study, MINAP and NAPCI generated 9.1% and 5.3% respectively. Patients who die in the ED are less likely to be recorded on MINAP, and only those patients receiving interventional cardiology are recorded in NAPCI.

Use of routine data has the potential to reduce the costs of conducting trials. The cost of the TASTE trial was reported as US$300,000, or approximately $50 per patient,[4] 2 per cent of the cost of a traditional randomised trial, but differs from the PARAMEDIC trial in that we did not use registry data to identify and recruit patients in the challenging and time-pressured setting of our-of-hospital cardiac arrest. In the West of Scotland Coronary Prevention (WOSCOPS) trial, data linkage reduced costs of long term follow up to less than one per cent of trial budget.[23] However, the time cost of linkage could be unrealistic for some trials. Linkage for the PARAMEDIC trial took up to three years from application to the trial team obtaining the data. It has been suggested that NHS Digital, who performed the linkage to HES for our study was overwhelmed with data linkage applications.[24] This may limit the usefulness of administrative data in trials with funder-imposed deadlines for completion.

For peer review only - http://bmjopen.bmj.com/site/about/guidelines.xhtml

Limitations:

Our study had several limitations. The matching reliability was suboptimal due to relaxed matching criteria (using range of event date), matching methods and potential issues of data quality and completeness, common to administrative data. Bohensky et al.[25] conducted an evidence synthesis of data linkage studies and identified factors such as sub-optimal or incomplete linkage leading to systematic bias. They considered the participant or population characteristics that can influence the validity and completeness of data linkage and may in turn lead to systematic bias in reporting. They reported variation in quality of data linkage across geographical/hospital sites, which could be due to high staff turnover or not sufficient resources allocated to the data collection and/or coding. We have not considered such variations in this study, but overall match quality was high in the matched cases.

Secondly, routine data were not fully available for all patients transported to hospital. Some patients were not included in the linkage as their data were not available in HES at the time of our data application. Although no substantial bias was shown, the generalisability of results could be limited. Several data fields were incomplete, for example, MINAP captures most ST elevation myocardial infarction (STEMI) cases but data for non-STEMI are less complete. We also cannot confirm how many patients required specialist care and should be included in non-HES datasets. Therefore, we were unable to assess and report the impact of unmatched cases in in the linkage to these registries.

Thirdly, we used the first matched admission without considering repeated or later admissions. We were therefore unlikely to fully describe patients' hospital pathway based on matched information.

Fourthly, our focus for the present study was on assessing the feasibility of using administrative data for purposes of follow-up. We did not assess the utility of administrative data to facilitate recruitment of trial patients since this was considered unrealistic in the clinical context of cardiac arrest.

Fifthly, we did not assess the financial cost of manual data collection at hospitals to compare with the cost of the use of registries in the trial linkage.

Recommendations

Based on our experience, we made the following recommendations to improve the use of data linkage in trials:

1. When planning a trial using linkage to administrative registries, careful planning is required to assess availability of the required data. Linkage to routine data in different jurisdictions or

For peer review only - http://bmjopen.bmj.com/site/about/guidelines.xhtml

multiple registries requires separate applications for data release and may be subject to data availability.

2. Trialists need to be mindful of prolonged processes for regulatory approvals, data release and validation. These processes may extend beyond trial funding.
3. Data linkage is a lengthy often unpredictable process in the application stage, possibly due to the restricted capacity of registries funded primarily to assess quality of care. Most registries in the NHS are funded as national audits and do not have sufficient resources for the timely processing of data sharing requests.
4. The quality of routinely collected data in the national registries may be inferior to that collected using traditional trial processes. Registry data are collected in high volume with limited resources and the validation process is unlikely to be as robust as in trials that are better resourced. Moreover, collected variables in registries are reviewed periodically and may change to reflect advances in clinical practice, which can impact on data completeness.
5. It is common for registries to charge a fee for data release, which should be costed in to trial budgets.

Conclusions

This study shows that it is feasible to track patients from the pre-hospital setting through to hospital admission using routinely available administrative datasets with a moderate to high degree of success. This may improve the efficiency and reduce the costs for longer-term follow-up in cardiac arrest trials.

For peer review only

Declaration of interests

GDP, RL, TQ, CDD and SG report grants from NIHR HTA Programme during the conduct of the study.

The other authors declare no competing interests.

Author's contributions

CS, JH, SG and GDP led on the data linkage. CJ analysed data. CJ, TQ and LG drafted the manuscript with input from the co-authors; RL, CS, JH, CDD, HP, MAS, NR, SJM, SG and GDP reviewed the manuscript. All authors approved the final version.

Acknowledgments

This is a summary of independent research partly funded by the National Institute for Health Research's (NIHR) Health Technology Assessment Programme (Grant Reference Number HTA-07/37/69). The views expressed are those of the author(s) and not necessarily those of the NHS, the NIHR, of the Department of Health. GDP received support as a NIHR Senior Investigator. MS is supported as an NIHR Doctoral Research Fellow. We thank the independent members of the Trial Steering Committee (Jon Nicholl, Helen Snooks, Fionna Moore, Alasdair Gray, Martyn Box, Father Neil Bayliss, and John Long) and the Data Monitoring Committee (Marion Campbell, Jerry Nolan, and Kathy Rowan). We acknowledge support from the OHCAO registry project that is funded by the British Heart Foundation and Resuscitation Council (UK).

Data sharing statement

No additional data sharing available.

For peer review only

For peer review only - http://bmjopen.bmj.com/site/about/guidelines.xhtml

References

1. Neumar RW, Shuster M, Callaway CW, et al. Part 1: Executive Summary: 2015 American Heart Association Guidelines Update for Cardiopulmonary Resuscitation and Emergency Cardiovascular Care. Circulation 2015;**132**(18 Suppl 2):S315-67. doi: 10.1161/CIR.0000000000000252
2. Whitehead L, Perkins GD, Clarey A, et al. A systematic review of the outcomes reported in cardiac arrest clinical trials: the need for a core outcome set. Resuscitation 2015;**88**:150-7. doi: 10.1016/j.resuscitation.2014.11.013
3. Lauer MS, Bonds D. Eliminating the "expensive" adjective for clinical trials. Am Heart J 2014;**167**(4):419-20. doi: 10.1016/j.ahj.2013.12.003
4. Frobert O, Lagerqvist B, Olivecrona GK, et al. Thrombus aspiration during ST-segment elevation myocardial infarction. N Engl J Med 2013;**369**(17):1587-97. doi: 10.1056/NEJMoa1308789
5. James S, Rao SV, Granger CB. Registry-based randomized clinical trials--a new clinical trial paradigm. Nat Rev Cardiol 2015;**12**(5):312-6. doi: 10.1038/nrcardio.2015.33
6. Lauer MS, D'Agostino RB, S.r. The randomized registry trial--the next disruptive technology in clinical research? N Engl J Med 2013;**369**(17):1579-81. doi: 10.1056/NEJMp1310102
7. Rao SV, Hess CN, Barham B, et al. A registry-based randomized trial comparing radial and femoral approaches in women undergoing percutaneous coronary intervention: the SAFE-PCI for Women (Study of Access Site for Enhancement of PCI for Women) trial. JACC Cardiovasc Interv 2014;**7**(8):857-67. doi: 10.1016/j.jcin.2014.04.007
8. Hofmann R, James SK, Svensson L, et al. DETermination of the role of OXygen in suspected Acute Myocardial Infarction trial. Am Heart J 2014;**167**(3):322-8. doi: 10.1016/j.ahj.2013.09.022
9. Perkins GD, Woollard M, Cooke MW, et al. Prehospital randomised assessment of a mechanical compression device in cardiac arrest (PaRAMeDIC) trial protocol. Scand J Trauma Resusc Emerg Med 2010;**18**:58. doi: 10.1186/1757-7241-18-58
10. Perkins GD, Lall R, Quinn T, et al. Mechanical versus manual chest compression for out-of-hospital cardiac arrest (PARAMEDIC): a pragmatic, cluster randomised controlled trial. Lancet 2015;**385**(9972):947-55. doi: 10.1016/S0140-6736(14)61886-9
11. Ji C, Lall R, Quinn T, et al. Post-admission outcomes of participants in the PARAMEDIC trial: A cluster randomised trial of mechanical or manual chest compressions. Resuscitation 2017;**118**:82-88. doi: 10.1016/j.resuscitation.2017.06.026
12. Marti J, Hulme C, Ferreira Z, et al. The cost-effectiveness of a mechanical compression device in out-of-hospital cardiac arrest. Resuscitation 2017;**117**:1-7. doi: 10.1016/j.resuscitation.2017.04.036
13. Rajagopal S, Kaye CR, Lall R, et al. Characteristics of patients who are not resuscitated in out of hospital cardiac arrests and opportunities to improve community response to cardiac arrest. Resuscitation 2016;**109**:110-15. doi: 10.1016/j.resuscitation.2016.09.014
14. Herrett E, Smeeth L, Walker L, et al. The Myocardial Ischaemia National Audit Project (MINAP). Heart 2010;**96**(16):1264-7. doi: 10.1136/hrt.2009.192328
15. Ludman PF, de Belder MA, McLenachan JM, et al. The importance of audit to monitor applications of procedures and improve primary angioplasty results. EuroIntervention 2012;**8 Suppl P**:P62-70. doi: 10.4244/EIJV8SPA11
16. Harrison DA, Brady AR, Rowan K. Case mix, outcome and length of stay for admissions to adult, general critical care units in England, Wales and Northern Ireland: the Intensive Care National Audit & Research Centre Case Mix Programme Database. Crit Care 2004;**8**(2):R99-111. doi: 10.1186/cc2834
17. Boyle MJ. The experience of linking Victorian emergency medical service trauma data. BMC Med Inform Decis Mak 2008;**8**:52. doi: 10.1186/1472-6947-8-52

For peer review only - http://bmjopen.bmj.com/site/about/guidelines.xhtml

18. Seymour CW, Kahn JM, Martin-Gill C, et al. Creating an infrastructure for comparative effectiveness research in emergency medical services. Acad Emerg Med 2014;**21**(5):599-607. doi: 10.1111/acem.12370
19. Mears GD, Rosamond WD, Lohmeier C, et al. A link to improve stroke patient care: a successful linkage between a statewide emergency medical services data system and a stroke registry. Acad Emerg Med 2010;**17**(12):1398-404. doi: 10.1111/j.1553-2712.2010.00925.x
20. Hettinger AZ, Cushman JT, Shah MN, et al. Emergency medical dispatch codes association with emergency department outcomes. Prehosp Emerg Care 2013;**17**(1):29-37. doi: 10.3109/10903127.2012.710716
21. Barry SJ, Dinnett E, Kean S, et al. Are routinely collected NHS administrative records suitable for endpoint identification in clinical trials? Evidence from the West of Scotland Coronary Prevention Study. PLoS One 2013;**8**(9):e75379. doi: 10.1371/journal.pone.0075379
22. Li G, Sajobi TT, Menon BK, et al. Registry-based randomized controlled trials- what are the advantages, challenges, and areas for future research? J Clin Epidemiol 2016;**80**:16-24. doi: 10.1016/j.jclinepi.2016.08.003
23. McCowan C. Using routinely collected clinical data to support clinical trials: a view from Scotland. Proceedings from the Clinical Trials Ontario 2015 Clinical Trials Conference 04-05 March 2015. http://www.ctontario.ca/cms/media/cto-2015-conference-speaker-presentation_colin-mccowan.pdf (accessed 29 November 2017).
24. Filippon J. Slow and costly access to anonymised patient data impedes academic research. BMJ 2015;**351**:h5087. doi: 10.1136/bmj.h5087

25. Bohensky MA, Jolley D, Sundararajan V, et al. Data linkage: a powerful research tool with potential problems. BMC Health Serv Res 2010;**10**:346. doi: 10.1186/1472-6963-10-346

For peer review only

For peer review only

For peer review only - http://bmjopen.bmj.com/site/about/guidelines.xhtml

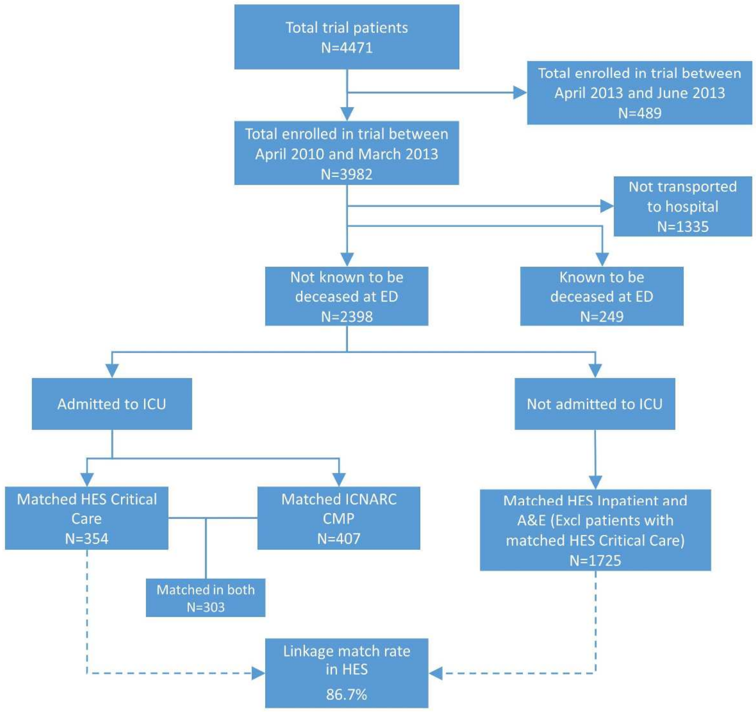

Figure 1: Linkage match rate and flowchart of patients retrieving HES or CMP data.

Legend: ICU, intensive care unit; ICNARC, Intensive Care National Audit and Research Centre; CMP, Case Mix Programme, HES, Hospital Episode Statistics; ED, Emergency Department; A&E, Accident and Emergency.

254x190mm (300 x 300 DPI)

Supplementary materials:

Supplementary Table 1: Summary of retrieved information in each linked dataset.

HES Inpatient		All
CSK operation	No	606 (79.6%)
	Yes	155 (20.4%)
Destination of discharge	The usual place of residence, including no fixed abode	192 (24.9%)
	Temporary place of residence when usually resident elsewhere	4 (0.5%)
	Repatriation from high security psychiatric hospital (from 1999-2000)	0 (0.0%)
	Repatriation from high security psychiatric hospital (from 1999-2000)	0 (0.0%)
	Penal establishment - court (from 1999-2000)	0 (0.0%)
	Penal establishment - police station (from 1999-2000)	0 (0.0%)
	Penal establishment - court and police station excluded (from 1999-2000 to 2006-07)	0 (0.0%)
	High security psychiatric hospital, Scotland (from 1999-2000)	0 (0.0%)
	NHS other hospital provider - high security psychiatric accommodation	0 (0.0%)
	NHS other hospital provider - medium secure unit (from 1999-2000)	32 (4.2%)
	NHS other hospital provider - ward for maternity patients or neonates	0 (0.0%)
	NHS other hospital provider - ward for patients who are mentally ill or have learning disabilities	0 (0.0%)
	NHS run nursing home, residential care home or group home	3 (0.4%)
	Local authority Part 3 residential accommodation - where care is provided (from 1996-97)	0 (0.0%)
	Local authority foster care, but not in Part 3 residential accommodation - where care is provided (from 1996-97)	0 (0.0%)
	LA home or care (1989-90 to 1995-96)	0 (0.0%)
	Non-NHS run hospital - medium secure unit (from 2003-04)	1 (0.1%)
	Non-NHS (other than local authority) run residential care home (from 1996-97 to 2003-04) and care home (from 2003-04)	3 (0.4%)
	Non-NHS (other than local authority) run nursing home (from 1996-97 to 2003-04)	0 (0.0%)
	Non-NHS run hospital	0 (0.0%)
	Non-NHS (other than local authority) run hospice	2 (0.3%)
	Non-NHS institution (1989-90 to 1995-96)	0 (0.0%)
	Not applicable	7 (0.9%)
	Not known	1 (0.1%)
	Died	522 (67.7%)
Method of discharge	Discharged on clinical advice or with clinical consent	234 (30.4%)
	Self discharged, or discharged by a relative or advocate	5 (0.6%)

For peer review only

For peer review only - http://bmjopen.bmj.com/site/about/guidelines.xhtml

	Discharged by a mental health review tribunal, the Home Secretary or a court	0 (0.0%)
	Baby was still born	0 (0.0%)
	Not applicable: patient still in hospital	10 (1.3%)
	Not known: a validation error	0 (0.0%)
	Died	522 (67.7%)
IMD 2004 index	Missing	84 (10.9%)
	Least deprived 10%	64 (8.3%)
	Less deprived 10-20%	43 (5.6%)
	Less deprived 20-30%	47 (6.1%)
	Less deprived 30-40%	35 (4.5%)
	Less deprived 40-50%	50 (6.5%)
	More deprived 10-20%	80 (10.4%)
	More deprived 20-30%	84 (10.9%)
	More deprived 30-40%	58 (7.5%)
	More deprived 40-50%	61 (7.9%)
	Most deprived 10%	165 (21.4%)
Hospital length of stay*		7.7 (20.3)
	**HES A&E**	**All**
A&E department type - A&E only	Emergency departments	1865 (96.8%)
	Consultant-led mono specialty accident and emergency service'	0 (0.0%)
	Other type of A&E. Excludes NHS walk-in centres	4 (0.2%)
	NHS walk-in centres	1 (0.1%)
	Not known	57 (3.0%)
A&E attendance disposal - A&E only	Admitted to hospital bed / became a lodged patient of the same health care provider	560 (29.1%)
	Discharged – follow-up treatment to be provided by general practitioner	5 (0.3%)
	Discharged – did not require any follow-up treatment	9 (0.5%)
	Referred to A&E clinic	13 (0.7%)
	Referred to fracture clinic	1 (0.1%)
	Referred to other outpatient clinic	1 (0.1%)
	Transferred to other healthcare provider	28 (1.5%)
	Referred to other healthcare professional	6 (0.3%)
	Left department before being treated	1 (0.1%)
	Left department having refused treatment	0 (0.0%)
	Other	5 (0.3%)
	Not known	2 (0.1%)
	Died in department	1296 (67.3%)
IMD 2004 index	Missing	7 (0.4%)
	Least deprived 10%	126 (6.5%)
	Less deprived 10-20%	95 (4.9%)
	Less deprived 20-30%	139 (7.2%)
	Less deprived 30-40%	120 (6.2%)

For peer review only - http://bmjopen.bmj.com/site/about/guidelines.xhtml

	Less deprived 40-50%	133 (6.9%)
	More deprived 10-20%	257 (13.3%)
	More deprived 20-30%	213 (11.1%)
	More deprived 30-40%	173 (9.0%)
	More deprived 40-50%	205 (10.6%)
	Most deprived 10%	459 (23.8%)
**HES Critical care**		**All**
Length of level 2 CC days		0.9 (1.7)
Length of level 3 CC days		4.4 (5.4)
Total length of CC days		6.6 (15.4)
**ICNARC CMP**		**All**
Length of level 0 ITU days		0 (0.3)
Length of level 1 ITU days		0.1 (0.6)
Length of level 2 ITU days		1.1 (4.0)
Length of level 3 ITU days		5.2 (9.4)
Total length of ITU days		6.5 (12.9)
Total length of ITU days in 30 days survived patients		10.5 (22.0)
Total length of ITU days in 30 days deceased patients		4.8 (4.5)
Days of alive and free of ITU stay in the first 28 days of cardiac arrest		6.9 (10.3)
Treatment withheld/withdrawn	Both withheld then withdrawn	23 (5.7%)
	Withheld	4 (1.0%)
	Withdrawn	133 (32.7%)
	Neither	247 (60.7%)
Organ donation	Heartbeating solid organ donor	13 (3.2%)
	No solid organs or tissues donated	199 (48.9%)
	Non-heartbeating solid organ donor	15 (3.7%)
	Tissue donor only	8 (2.0%)
**NICOR MINAP**		**All**
Admission Diagnosis	Definite myocardial infarction	126 (69.2%)
	Acute coronary syndrome	30 (16.5%)
	Chest pain cause	4 (2.2%)
	Other initial diagnosis	22 (12.1%)
Admission Ward	Missing	1 (0.5%)
	Cardiac care unit	94 (51.6%)
	Acute admissions unit	6 (3.3%)
	General medical ward	1 (0.5%)
	Intensive therapy unit	69 (37.9%)
	Other	9 (4.9%)
	Cardiac ward (non CCU)	1 (0.5%)
	Stepdown ward	0 (0.0%)
	Unknown	0 (0.0%)
	Died in A&E	1 (0.5%)

For peer review only - http://bmjopen.bmj.com/site/about/guidelines.xhtml

Initial Reperfusion Treatment	Missing	3 (2.2%)
	None	18 (13.1%)
	Thrombolytic treatment	0 (0.0%)
	pPCI in house	116 (84.7%)
	Referred for consideration for pPCI elsewhere	0 (0.0%)*
	Unknown	0 (0.0%)
Procedure performed	Missing	30(21.9%)
	No angiogram	2 (1.5%)
	Angiogram but no PCI	9 (6.6%)
	Angiogram and PCI	96 (70.1%)
	Unknown	0 (0.0%)
Coronary angiography	Missing	1(2.9%)
	Protocol driven investigation performed in this hospital	12(35.3%)
	Symptom driven investigation performed in this hospital	7 (20.6%)
	Protocol driven investigation performed at another hospital	0 (0.0%)
	Symptom driven investigation performed at another hospital	0 (0.0%)
	Planned after discharge	0 (0.0%)
	Not applicable	9 (6.6%)
	Patient refused	1 (2.9%)
	Not performed	5 (14.7%)
	Unknown	0 (0.0%)
Coronary intervention	Missing	6 (17.6%)
	Percutaneous coronary intervention	9 (26.5**%)**
	CABG	0 (0.0%)
	PCI planned after discharge	0 (0.0%)
	CABG planned after discharge	1(2.9%)
	Not applicable	5 (14.7%)
	Patient refused	1 (2.9%)
	Not performed or arranged	11 (32.4%)
	Unknown	0 (0.0%)
Assessment at non-interventional centre	Missing	5 (2.7%)
	No contact with a non interventional hospital	126 (69.2%)
	Patient remains in ambulance	0 (0.0%)
	A&E	30 (16.5%)
	Acute assessment unit	2 (1.1%)
	CCU / cardiac facility	1 (0.5%)
	Self referral	0 (0.0%)
	Already in hospital	2 (1.1%)
	Other	0 (0.0%)
	Unknown	16 (8.8%)
Assessment at interventional centre	Missing	59 (32.4%)
	Assessed in A&E	68 (37.4%)
	Acute assessment unit	1 (0.5%)
	CCU / cardiac facility	26 (14.3%)

For peer review only - http://bmjopen.bmj.com/site/about/guidelines.xhtml

	Catheter laboratory	28 (15.4%)
	Already in hospital	0 (0.0%)
	Unknown	0 (0.0%)
**NICOR PCI**		**All**
Cardiopulmonary resuscitation (CPR)	Missing	29 (28.7%)
	No	72 (71.3%)
	Yes	0 (0.0%)
Coronary artery bypass grafting (CABG)	Missing	0 (0.0%)
	No	101 (100%)
	Yes	0 (0.0%)
Ventilation	Missing	101 (100%)*
	No	0 (0.0%)
	Yes	0 (0.0%)
Hypothermia	Missing	101 (100%)*
	No	0 (0.0%)
	Yes	0 (0.0%)
Percutaneous coronary intervention (PCI)	Missing	101 (100%)
	No	0 (0.0%)
	Yes	0 (0.0%)
	No	0 (0.0%)
	Yes	0 (0.0%)

Note: *: Variables were added to the dataset after the linkage was performed.

For peer review only - http://bmjopen.bmj.com/site/about/guidelines.xhtml

Supplementary Table 2: Summary of demographic and cardiac arrest event characteristics in trial patients with and without matched HES inpatient, Critical care and A&E data

		HES matched N=2079	HES unmatched N=319	p value
Age (year)		73.6 (21.9)	71.8 (24.4)	0.151
Sex	Male	1315 (63.3%)	215 (67.4%)	0.151
	Female	764 (36.8%)	104 (32.6%)	
Initial aetiology	Presumed cardiac	1786 (85.9%)	272 (85.3%)	0.864
	Respiratory	146 (7.0%)	27 (8.5%)	
	Submersion	8 (0.4%)	1 (0.3%)	
	Other	74 (3.6%)	9 (2.8%)	
	Unknown	65 (3.1%)	10 (3.1%)	
Initial rhythm	VF	624 (30.0%)	99 (31.0%)	0.468
	VT	20 (1.0%)	1 (0.3%)	
	PEA	613 (29.5%)	83 (26.0%)	
	Asystole	727 (35.0%)	118 (37.0%)	
	Unknown	95 (4.6%)	18 (5.6%)	
Location	Home	1610 (77.4%)	206 (64.6%)	<0.001
	Public place	333 (16.0%)	89 (27.9%)	
	Other	136 (6.5%)	24 (7.5%)	
Witness	Not witnessed	508 (24.5%)	60 (18.8%)	0.027
	By bystander	961 (46.3%)	170 (53.3%)	
	By EMS	413 (19.9%)	60 (18.8%)	
	By Non-EMS healthcare	64 (3.1%)	4 (1.3%)	
	Unknown	132 (6.4%)	25 (7.8%)	
Cardiopulmonary resuscitation (CPR) by Bystander	No	1104 (53.1%)	156 (48.9%)	0.376
	Yes	862 (41.5%)	144 (45.1%)	
	Unknown	113 (5.4%)	19 (6.0%)	
Response time (minute)†		6.1 (4.3)	7.2 (5.1)	<.0001
Survival at 30days	Alive	224 (10.8%)	38 (11.9%)	0.544
	Deceased	1855 (89.2%)	281 (88.1%)	
ROSC at hospital transfer	ROSC	790 (38.0%)	125 (39.2%)	<0.001
	CPR in progress	1204 (57.9%)	135 (42.3%)	
	Unknown	85 (4.1%)	59 (18.5%)	
EQ5d at 3 months		70.0 (20.0)	75.0 (20.0)	0.970
EQ5d at 12 months		80.0 (30.0)	72.5 (26.0)	0.368
SF12 mental health at 3 months		50.4 (16.8)	45.4 (23.0)	0.690
SF12 physical health at 3 months		40.8 (14.2)	44.1 (15.0)	0.533
SF12 mental health at 12 months		51.0 (14.5)	44.1 (10.5)	0.118
SF12 physical health at 12 months		45.2 (17.4)	38.4 (13.9)	0.059
HADS Anxiety at 12 months		5.0 (7.0)	6.5 (5.0)	0.112

For peer review only - http://bmjopen.bmj.com/site/about/guidelines.xhtml

HADS depression at 12 months	4.0 (5.0)	6.0 (4.5)	0.163
MMSE at 12 months	29.0 (3.0)	29.0 (2.0)	0.648
PTSD at 12 months	27.0 (17.0)	33.0 (19.0)	0.280

Note: Continuous variables were shown as median (Interquartile range) and categorical variables were shown as n (percentage). †: response time was from 999 call to EMS arrival at scene.

For peer review only

For peer review only - http://bmjopen.bmj.com/site/about/guidelines.xhtml

Supplementary Table 3: Summary of demographic and cardiac arrest event characteristics in patients with matched HES Critical care only, CMP only and both.

		HES Critical Care only	ICNARC CMP only	Critical care & CMP	p value
Age (year)		68.3 (24.6)	64.8 (28.2)	65.5 (21.2)	0.684
Sex	Male	36 (70.6%)	66 (63.5%)	208 (68.7%)	0.556
	Female	15 (29.4%)	38 (36.5%)	95 (31.4%)	
Aetiology	Presumed cardiac	45 (88.2%)	84 (80.8%)	247 (81.5%)	0.554
	Respiratory	1 (2.0%)	12 (11.5%)	25 (8.3%)	
	Submersion	0 (0.0%)	1 (1.0%)	1 (0.3%)	
	Other	3 (5.9%)	6 (5.8%)	20 (6.6%)	
	Unknown	2 (3.9%)	1 (1.0%)	10 (3.3%)	
Location	Home	37 (72.6%)	68 (65.4%)	209 (69.0%)	0.516
	Public place	14 (27.5%)	30 (28.9%)	79 (26.1%)	
	Other	0 (0.0%)	6 (5.8%)	15 (5.0%)	
Witness	No	11 (21.6%)	26 (25%)	69 (22.8%)	0.981
	By bystander	29 (56.9%)	59 (56.7%)	174 (57.4%)	
	By EMS	7 (13.7%)	13 (12.5%)	32 (10.6%)	
	By Non-EMS healthcare	1 (2.0%)	2 (1.9%)	9 (3.0%)	
	Unknown	3 (5.9%)	4 (3.9%)	19 (6.3%)	
Cardiopulmonary resuscitation	No	24 (47.1%)	45 (43.3%)	136 (44.9%)	0.802
	Yes	24 (47.1%)	52 (50.0%)	155 (51.2%)	
	Unknown	3 (5.9%)	7 (6.7%)	12 (4.0%)	
Rhythm	VF	26 (51%)	44 (42.3%)	153 (50.5%)	0.437
	VT	2 (3.9%)	1 (1.0%)	4 (1.3%)	
	PEA	10 (19.6%)	25 (24.0%)	55 (18.2%)	
	Asystole	11 (21.6%)	26 (25.0%)	80 (26.4%)	
	Unknown	2 (3.9%)	8 (7.7%)	11 (3.6%)	
Response time (minute)†		6.6 (4.4)	6.7 (4.7)	6.0 (3.9)	0.034
Survival at 30days	Alive	19 (37.3%)	31 (29.8%)	93 (30.7%)	0.606
	Deceased	32 (62.8%)	73 (70.2%)	210 (69.3%)	
ROSC at hospital transfer	ROSC	42 (82.4%)	79 (76%)	241 (79.5%)	0.294
	CPR in progress	7 (13.7%)	14 (13.5%)	47 (15.5%)	
	Unknown	2 (3.9%)	11 (10.6%)	15 (5.0%)	
EQ5d at 3 months		67.5 (24.0)	75.5 (16.0)	70.0 (25.0)	0.838
EQ5d at 12 months		80.0 (14.0)	74.0 (20.0)	80.0 (25.0)	0.298
SF12 mental health at 3 months		48.4 (11)	48.3 (19.3)	49.3 (16.5)	0.794
SF12 physical health at 3 months		38.9 (13.2)	39.2 (13)	41.1 (14.2)	0.836
SF12 mental health at 12 months		51.6 (8)	43 (14.3)	47.8 (15.2)	0.543
SF12 physical health at 12 months		42.9 (21)	38.8 (14.6)	46.7 (15.6)	0.235

For peer review only - http://bmjopen.bmj.com/site/about/guidelines.xhtml

HADS Anxiety at 12 months	5.0 (5.0)	9.5 (6.5)	6.0 (6.0)	0.078
HADS depression at 12 months	4.0 (3.0)	6.0 (5.5)	5.0 (8.0)	0.368
MMSE at 12 months	28.0 (3.0)	29.0 (3.0)	29.0 (3.0)	0.717
PTSD at 12 months	30.0 (8.5)	40.0 (23.0)	29.0 (15.0)	0.631

Note: Continuous variables were shown as median (Interquartile range) and categorical variables were shown as frequency (percentage). †: response time is from 999 call to EMS arrival at scene.

For peer review only

BMJ Open

Feasibility of data linkage in the PARAMEDIC Trial: a cluster randomised trial of mechanical chest compression in out of hospital cardiac arrest

Journal:	*BMJ Open*
Manuscript ID	bmjopen-2018-021519.R1
Article Type:	Research
Date Submitted by the Author:	16-May-2018

Complete List of Authors:	Ji, Chen; University of Warwick, Warwick Clinical Trials Unit<br>Quinn, Tom; St George's Univesity of London & Kingston University, Faculty of Health, Social Care & Education<br>Gavalova, Lucia; St George's Univesity of London & Kingston University, Faculty of Health, Social Care & Education<br>Lall, Ranjit; University of Warwick, Warwick Clinical Trials Unit<br>Scomparin, Charlotte; University of Warwick, Warwick Clinical Trials Unit<br>Horton, Jessica; University of Warwick, Warwick Clinical Trials Unit<br>Deakin, Charles; Southampton University Hospital, Shackleton Department of Anaesthetics<br>Pocock, Helen; South Central Ambulance Service NHS Foundation Trust<br>Smyth, Michael; University of Warwick, Warwick Clinical Trials Unit<br>Rees, Nigel; Welsh Ambulance Service NHS Trust<br>Brace-McDonnell, Samantha; University of Warwick, Warwick Clinical Trials Unit<br>Gates, Simon; University of Birmingham , Cancer Research UK Clinical Trials Unit, Institute of Cancer and Genomic Sciences; University of Warwick, Warwick Clinical Trials Unit<br>Perkins, Gavin; University of Warwick, Warwick Clinical Trials Unit; Heart of England NHS Foundation Trust

**Primary Subject Heading**:	Research methods
Secondary Subject Heading:	Research methods, Epidemiology
Keywords:	Adult cardiology < CARDIOLOGY, Clinical trials < THERAPEUTICS, Data linkage, Cardiac arrest

SCHOLARONE™ Manuscripts

Feasibility of data linkage in the PARAMEDIC Trial: a cluster randomised trial of mechanical chest compression in out of hospital cardiac arrest

Chen Ji[1] (C.Ji.3@warwick.ac.uk)

Tom Quinn[2] (T.Quinn@sgul.kingston.ac.uk)

Lucia Gavalova[2] (L.Gavalova@sgul.kingston.ac.uk)

Ranjit Lall[1] (R.Lall@warwick.ac.uk)

Charlotte Scomparin[1] (C.Scomparin@warwick.ac.uk)

Jessica Horton[1] (jessicahorton79@hotmail.com)

Charles D Deakin[3,4] (charlesdeakin@doctors.org.uk)

Helen Pocock[4] (Helen.Pocock1@nhs.net)

Michael A Smyth[1] (M.A.Smyth@warwick.ac.uk)

Nigel Rees[5] (Nigel.Rees5@wales.nhs.uk)

Samantha J Brace-McDonnell[1,6] (S.Brace-Mcdonnell@warwick.ac.uk)

Simon Gates[1,7] (S.Gates@bham.ac.uk)

Gavin D Perkins[1,6] (G.D.Perkins@warwick.ac.uk)

1. Warwick Clinical Trials Unit, University of Warwick, Coventry, UK

2. Faculty of Health, Social Care and Education, Kingston University and St George's, University of London, London, UK

3. NIHR Southampton Respiratory Biomedical Research Unit, University Hospital Southampton NHS Foundation Trust, Southampton, Hampshire, UK

4. South Central Ambulance Service NHS Foundation Trust, Otterbourne, UK

5. Welsh Ambulance Service NHS Trust, Cardiff, UK

6. Heart of England NHS Foundation Trust, Birmingham, UK

7. Cancer Research UK Clinical Trials Unit, University of Birmingham, Birmingham, UK

Correspondence to

Professor Gavin Perkins; University of Warwick, Tel: +44 (0)24761 50925; G.D.Perkins@warwick.ac.uk

For peer review only

Word count

Abstract: 280

Main body: 3554 (excluding Tables/Figures/titles/notes/legends)

For peer review only

For peer review only - http://bmjopen.bmj.com/site/about/guidelines.xhtml

Abstract

Objectives: There is considerable interest in reducing the cost of clinical trials. Linkage of trial data to administrative datasets and disease-specific registries may improve trial efficiency, but has not been reported in resuscitation trials conducted in the UK. To assess the feasibility of utilising national administrative and clinical datasets to follow up patients transported to hospital following attempted resuscitation in a cluster randomised trial of a mechanical chest compression device in out of hospital cardiac arrest (OHCA).

Methods: Hospital data on trial participants were requested from Hospital Episode Statistics (HES); the Intensive Care National Audit and Research Centre (ICNARC); and Myocardial Ischaemia National Audit Project (MINAP) and National Audit of Percutaneous Coronary Intervention (NAPCI), using unique patient identifiers. Linked data were received between June 2014 and June 2015.

Results: Of 4471 patients randomised in the PARAMEDIC trial, 2398 (53.6%) were not known to be deceased at emergency department arrival and were eligible for linkage. We achieved an overall match rate of 86.7% in the combined HES A&E, inpatient and Critical care dataset, with variable match rates (4.2-80.4%) in individual datasets. Patient demographics, cardiac arrest related characteristics and major outcomes were predominantly similar between HES matched and unmatched groups, in the linkage apart from location, response time and ROSC at handover.

Conclusions: This study shows that it is feasible to track patients from the pre-hospital setting through to hospital admission using routinely available administrative datasets with a moderate to high degree of success. This approach has the potential to complement the trial data with the demographic and clinical management information about the studied cohort, as well as to improve the efficiency and reduce the costs of follow-up in cardiac arrest trials.

ISRCTN08233942.

For peer review only - http://bmjopen.bmj.com/site/about/guidelines.xhtml

Strengths and limitations of this study

- First study evaluating the supplement of routinely collected administrative data in a cardiac arrest trial in the UK.
- Data linkage was made to different UK national registries.
- The matching reliability was suboptimal due to relaxed matching criteria, matching method and possible data quality issues.
- Routine data were not fully available for all trial patients transported to hospital.
- The findings of our study are not generalisable to facilitate trial recruitment since it was considered unrealistic in the clinical context of cardiac arrest.

For peer review only

Background

Well conducted and reported randomised controlled trials (RCTs) are considered the gold standard in evaluation of new or established clinical interventions. In cardiac arrest resuscitation science, only a small minority (1%) of contemporary international guideline recommendations are based on the highest level of evidence from more than one RCT, meta-analysis of high quality RCTs, or RCTs corroborated by high quality registry studies.[1] High quality trials to address outcomes of interest to patients following cardiac arrest (e.g. long term survival, neurocognitive status and disability)[2] are complex, labour intensive and expensive to perform. Many studies in cardiac arrest are therefore too small or inadequately conducted (with a predominance of observational studies which are prone to bias) to provide reliable estimates of treatment effect or harm to patients. Consequently, for the majority of resuscitation interventions, there is a paucity of high quality evidence. Funders (typically government agencies) have called for proposals for low-cost, more efficient trials.[3]

Traditional trial methods of patient tracking and data access in individual hospitals is challenging with limited resources. Cardiovascular medicine has attempted to improve the efficiency of the trial design by pioneering the concept of registry-based randomised trials, using clinical quality registries and administrative datasets. In the Thrombus Aspiration during ST-Segment Elevation Myocardial Infarction (TASTE) trial, undertaken in Sweden, both patient enrolment and follow up were conducted using the Swedish Web System for Enhancement and Development of Evidence-based Care in Heart Disease Evaluated According to Recommended Therapies (SWEDEHEART) registry.[4] On publication, this registry-based trial was hailed as the '*next disruptive technology*' in clinical research, and as a new clinical trial paradigm.[5 6] Subsequent registry-based trials have been reported in a comparison of radial versus femoral access in women undergoing percutaneous coronary intervention in the United States,[7] and of supplemental oxygen versus ambient air in patients with suspected acute myocardial infarction in Sweden.[8]

To our knowledge, however, there are no reports of registry-based randomised trials in resuscitation science. However, should accessing registry data to ascertain outcomes in a prehospital cardiac arrest trial (e.g. length of stay/patient pathways/survival status) to be feasible, this could be one way of significantly improving efficiency and reducing costs of conducting high quality randomised trials in resuscitation.

In the PARAMEDIC trial, the in-hospital data collection process was complex, expensive and labour intensive, with research paramedics visiting multiple hospitals across large geographical areas to extract data from hospital records. Patients transported to hospital following resuscitation from cardiac arrest follow multiple clinical pathways depending on their clinical status and treatments. As

For peer review only - http://bmjopen.bmj.com/site/about/guidelines.xhtml

hospital data are routinely collected and managed by national registries, utilising these registries could save resources and time in the in-hospital data collection and potentially reduce the burden on patients and relatives in the sensitive period following cardiac arrest.

This paper reports our assessment of the feasibility of linking data collected for the purposes of patient follow up in a pragmatic, cluster randomised controlled trial of a mechanical chest compression device undertaken in the United Kingdom (UK) prehospital setting, with large national administrative and specialist registries.

Methods

The PARAMEDIC trial examined the effectiveness of LUCAS-2, a mechanical chest compression device, in 4471 patients with out of hospital cardiac arrest (OHCA). The study was a cluster randomised trial whereby emergency medical service (EMS) vehicles were randomised to carry the LUCAS-2 device (intervention) or not (control). Full details of the trial protocol have been published previously.[9] In summary, adults with OHCA where resuscitation was attempted by EMS personnel and attended by a trial vehicle were included. Patients with traumatic cardiac arrest or suspected to be pregnant were excluded. Trial recruitment ran from April 15, 2010 to June 10, 2013. We have previously reported primary outcome (30-day survival),[10] secondary outcomes,[11] an economic analysis[12] and characteristics of patients who were not resuscitated.[13]

Data sources

The PARAMEDIC trial utilised four sources of data that were linked to the trial dataset: UK National Health Service (NHS) Hospital Episodes Statistics (HES), Myocardial Ischaemia National Audit Project (MINAP),[14] National Audit of Percutaneous Coronary Interventions (NAPCI),[15] and Case Mix Programme (CMP)[16] to obtain data on hospital stay and treatment or procedures that trial patients received in hospital.

We used the MINAP, NAPCI and CMP data for the health economic analysis[12] and long-term post admission outcomes[11] and to validate the hospital length of stay or stay in the intensive care (secondary outcomes for the efficacy part of the trial), and also to gain insight into the specifics of the treatment or procedures that trial patients received during their hospital stay. Characteristics of the registries are summarised in Table 1.

Table 1: Characteristics of registries, participation and case ascertainment.

Registry/Dataset	Source	Description	Participation and case ascertainment* during the trial period

For peer review only - http://bmjopen.bmj.com/site/about/guidelines.xhtml

Paramedic trial	Warwick Clinical Trials Unit	Trial patient cohort that survived admission to a hospital	n/a
Hospital Episode Statistics (HES)	NHS Digital (NHSD)	Collection of information on all NHS hospital inpatients, Accident and Emergency (A&E), critical care and outpatients which enables health care providers to be paid according to their levels of activity.	All hospitals Case ascertainment 100%
Case Mix Programme (CMP)	Intensive Care National Audit and Research Centre (ICNARC)	Audit of patient outcomes from all adult, general critical care units in England, Wales and Northern Ireland. Other specialist units, including neurosciences, cardiac and high dependency units, also participate	Over 90% of critical care units<br><br>Case ascertainment not reported
Myocardial Ischaemia National Audit Project (MINAP)	National Institute for Cardiovascular Outcomes Research (NICOR)	National audit of patients with acute coronary syndrome admitted to all hospitals in England, Wales and Northern Ireland. Data are collected prospectively at each hospital by secure electronic system, electronically encrypted and transferred online to a central database	All hospitals<br><br>Case ascertainment not reported
NAPCI (National Audit of Percutaneous Coronary Interventions)	National Institute for Cardiovascular Outcomes Research (NICOR)	National audit of all PCI procedures from NHS and non-NHS hospitals in the United Kingdom.	All hospitals<br><br>Case ascertainment 97%

Note: *: Case ascertainment – Rate (e.g. %) of eligible cases included in a registry/database.

Patient population

Patients (denominator) for this linkage study were patients from the PARAMEDIC trial who were transported to hospital by EMS and not known to be deceased (i.e. documented as alive or unknown status) on arrival at the emergency department (ED).

Since NHS Digital, responsible for HES, only provides annual data up to 1st April each year, no data on trial patients recruited on or after 1st April 2013 had any HES data returned for this data request. We therefore limited our analysis of the linked registry data to patients recruited to the PARAMEDIC trial between April 2010 and March 2013.

Study approvals

The PARAMEDIC trial was approved by the Coventry Research Ethics Committee (reference 09/H1210/69) and sponsored by the University of Warwick, UK. The study was conducted in accordance with the principles of Good Clinical Practice and the Mental Capacity Act (2005). Specific approval for access to personal data without consent and the data linkage reported in this paper was obtained from the Confidentiality Advisory Group, part of the Health Research Authority (reference:

For peer review only

For peer review only - http://bmjopen.bmj.com/site/about/guidelines.xhtml

ECC 2-02 (c)/2011). At the time of the study this activity was undertaken by the National Information Governance Board for Health and Social Care Ethics and Confidentiality Committee.

Patient and public involvement

Patient and public representatives (PPR) were invited to the Trial Steering Committee meetings during the development and conduct of the main trial. They agreed with the data collection via linkage to reduce the burden on patients and relatives. They were regularly informed of this study and other trial outputs. The results of this study will be disseminated in different ways, including presentation on the publicly accessible trial webpage.

Data linkage procedure

Data access applications were submitted to national administrative and disease registries between 2012-2014 to request patient case mix and clinical variables (Supplementary Table 1). The following patient identifiers were sent to the NHSD, ICNARC and NICOR to identify their clinical records: trial number, cardiac arrest date, ambulance service case number, 999 call time, hospital name, hospital arrival time, hospital handover time, patient name, NHS number, home address and postcode. The trial data were linked to the two NICOR datasets (MINAP and NAPCI) on two separate occasions by a different member of NICOR staff, which reassuringly generated the same results. Extracted anonymous data were encrypted and sent back to the trial team between June 2014 and June 2015.

Linked data may contain multiple, non-event related hospital records within the requested linkage period. We firstly used patient cardiac arrest (trial event) date to identify the records with exactly matched admission/visit date in the respective data sources. However, event and admission dates could be different due to potential data definition discrepancies. For instance, a trial event could occur before midnight and the patient was admitted to hospital after midnight. Therefore, we relaxed the date match criterion to a 5-day range (date of cardiac arrest with +/- 2 days). A matched record was redefined as if the admission/visit date falls in the range. We considered the range would be sufficiently large to mitigate against any date discrepancies in different sources and also be reasonably small to reduce the chance of mismatch in the case of early re-admission. Where multiple records could be matched to a single trial event in the same routine dataset, separate rules were used to extract the retrieved information: 1) where a patient had multiple episodes in HES, only the one with recorded death or discharge date was retained. If a patient had not been discharged from hospital, the episode with latest ward admission date was used. 2) Where multiple admissions to ICU were recorded in CMP, only the first ICU admission was linked to a trial event. 3) Since the MINAP dataset provided to us by NICOR only contained year and month of admission, only the earliest admission was used. 4) Only the first procedure was included for the linkage to the

For peer review only - http://bmjopen.bmj.com/site/about/guidelines.xhtml

NAPCI registry data, since patients can have more than one interventional procedure (and thus another record) during the index admission.

Data linkage rate

For HES data, we developed the linkage and match rate for linked and matched (or correctly linked) cases as follows:

$$\text{HES linkage rate} = \frac{\textbf{N of patients with linked HES inpatient, Critical care or A\&E data}}{\textbf{N of patients not known to be deceased at ED}}$$

$$\text{HES match rate} = \frac{\textbf{N of patients with matched (correctly linked) HES inpatient, Critical care or A\&E data}}{\textbf{N of patients not known to be deceased at ED}}$$

Similar equations were used to determine the rates for each of the datasets i.e. MINAP, NAPCI and CMP. As we were not able to confirm which patient should actually be collected in these datasets, we employed same denominator used in the above equations.

Data linkage quality

Match rank is an indicator used in HES to show the confidence of match: 1 suggests the best match and 8 the worst. Level 1-3 appear to be of high quality as cases are matched based on a combination of unique NHS number and data of date of birth, sex and home postcode. The quality of linkage in matched HES was therefore summarised on the basis of percentage of level 1-3.

Data representativeness

Data representativeness was assessed in two comparisons. The first comparison intended to assess whether the patients with correctly linked (i.e. matched) HES data could be representative of the trial population. It was carried out in patients with and without matched HES inpatient, Critical Care or A&E data (comparison 1). The second comparison intended to assess the difference between two critical care data sources. We were not able to compare data from these two sources directly as some patient care data were collected in both databases. Hence, we split the patients by their linked data sources and made the comparison between patients with HES Critical Care only, with CMP data only and with both HES Critical Care and CMP data (comparison 2).

For both comparisons, we compared patient and event characteristics between the datasets. Continuous variables were compared using Mann-Whitney test in comparison 1 and Kruskal-Wallis test in comparison 2. Categorical variables were compared using Chi-square test. A two-sided p

For peer review only

For peer review only - http://bmjopen.bmj.com/site/about/guidelines.xhtml

value <0.05 was considered statistically significant. All analyses were conducted in SAS v9.3 (Cary, NC, USA).

Data security and destruction

We followed the Warwick Clinical Trials Unit Standard Operating Procedures (SOPs) for data storage, transfer, and data sharing. The data were retained and destroyed in accordance with relevant regulations and the University of Warwick's Data Sharing Agreements.

Results

In the PARAMEDIC trial, 2695 patients were transported to hospital and not known to be deceased at ED. Of these 2398 (89.0%) were recruited between April 2010 and March 2013 and were therefore included in this study (referred to as "linkage patients"). The data requests to NHSD, ICNARC and NICOR retrieved different numbers of patient clinical records.

Summary of the linkage

The flow chart of the linkage to HES is shown in Figure 1. The linkage patients were grouped into ICU admitted (patients with matched HES Critical care data) and not admitted (patients with other matched HES data). Meanwhile, patients with matched CMP data were also summarised in the flowchart. This presented a comparison between CMP and HES Critical Care. 303 patients were matched in both CMP and HES Critical Care. Overall, the linkage to HES data achieved a match rate of 86.7% (2079 of 2398) with allowed variation in dates (date of cardiac arrest with +/- 2 days), slightly improved from the use of exact date match approach (84.1%).

Linkage quality was high in matched cases: level 1-3 accounted for 97.9%. In unmatched cases, 91.5% (292 of 319) had no linked HES data and the rest, while linked with non-trial even related data, had a good match rank (<=3).

The summary of linkage and match rate in each dataset are shown in Table 2. All datasets contained multiple linked records, indicating some patients had been linked to multiple admissions with possible multiple episodes. Among the 2398 linkage patients, individual match rate varied depending on the hospitalisation stage and received treatments. HES A&E had the highest individual match rate (80.4%). In the patients admitted to ICU, CMP provided 53 more matched patients with a lower proportion of unmatched data in linked patients compared to HES Critical Care.

Figure 1: Linkage match rate and flowchart of patients retrieving HES or CMP data.

For peer review only - http://bmjopen.bmj.com/site/about/guidelines.xhtml

Table 2: Summary of linked PARAMEDIC trial patients to the respective registry databases

Data source	Dataset	Number of linked records	Number of linked patients (linkage rate)*	Number of matched patients (match rate)*
NHSD	HES Inpatient	12875	1617 (67.4%)	771 (32.2%)
	HES Critical care	545	433 (18.1%)	354 (14.8%)
	HES A&E	6434	2186 (91.2%)	1927 (80.4%)
	Overall	19854	2277 (95.0%)	2079 (86.7%)
ICNARC	CMP	435	410 (17.1%)	407 (17.0%)
NCAP	MINAP	244	218 (9.1%)	182 (7.6%)
	PCI	153	128 (5.3%)	101 (4.2%)

Note: *: Percentage is calculated using the number in the column divided by 2398 linkage patients.

A summary of retrieved information for each linked dataset as well as the degree of data missingness for each field is available in the online supplementary materials. In Supplementary Table 2, the trial patients that had not been matched to the HES records were similar to those that with matched records in age (mean age 71.8 and 73.6 respectively), male (67.4% and 63.3% respectively were male). They were also similar between groups in initial cardiac arrest aetiology where most were of cardiac origin (85.3% and 85.9% respectively) and in initial rhythm (shockable rhythm 31.0% and 31.3%). Patients with unmatched data were more likely to have had a cardiac arrest in a public place (27.9%) compared with of those with matched records (16%), witnessed by bystander (53.3% versus 46.3%) and had longer EMS response time (7.2 versus 6.1 minutes). Supplementary Table 3 illustrates the comparison of demographic and event characteristics of patients with matched HES Critical Care and CMP data. Characteristics were similar in all three groups, except for a significant difference in the EMS response time.

Discussion

This study aimed to demonstrate the feasibility of collecting trial outcome data during patient follow up in a prehospital cardiac arrest trial via linkage to national registries. We achieved an overall match rate of 86.7% in 2398 patients using HES data. The data linkage provided important administrative and additional clinical data that allowed extended analyses of the intervention effect and provided more details of patient journey in the trial. We also evaluated the representativeness of retrieved HES and CMP data by comparing patient and trial event characteristics. No substantial difference

For peer review only - http://bmjopen.bmj.com/site/about/guidelines.xhtml

was found in patients with and without matched HES inpatient, Critical Care or A&E data, as well as in patients with matched HES Critical Care only, CMP only and both datasets.

This was the first study evaluating the supplement of routinely collected administrative data in a cardiac arrest trial in the UK. Our match rate was in line with observational studies linking EMS data to hospital records,[17-20] and data validation studies.[21] Our experience suggests it is feasible to obtain relevant data from administrative databases in a cardiac arrest trial. In addition to the high match rate reported in this paper, the matched data are deemed to be sufficiently representative of the trial population. The comparison between patients with and without matched HES showed low level of imbalance of event characteristics. We have found similar results in the matched ICU data.

The unmatched cases were likely to be associated with missing or inaccurate data. Data quality could be at increased risk due to the challenging circumstances of cardiac arrest and complexity of patient handling following hospital arrival. In addition, routine data in the chosen registries are not systematically adjudicated. Lack of clinical engagement may compromise the case ascertainment and data quality,[22] leading to suboptimal linkage. NHS Digital employs deterministic and probabilistic methods in the data linkage. The latter calculates probability weight based on combinations of linkage variables and determines linkage based on a cut-off threshold. Although this method largely improves the linkage, it could incorrectly link record pairs and miss valid ones, undermining the reliability of linkage.

Linkage to individual routine datasets resulted in variable match rates. HES A&E generated the highest rate of 80.4% as most patients were taken by EMS to ED for assessment before being admitted to specialist hospital units. Other rates reflected the proportion of specific groups of patients in the linkage. The CMP, MINAP and NAPCI registries are focused on selected patients with a specific diagnosis and/or requiring specialist care, reflected in strict inclusion and exclusion criteria; for example MINAP comprises data on patients with suspected and/or confirmed acute coronary syndrome, NAPCI on interventional cardiology whilst CMP registry collects data on patients admitted to critical care/intensive care units within any given hospital. In this study, MINAP and NAPCI generated 9.1% and 5.3% respectively. Patients who die in the ED are less likely to be recorded on MINAP, and only those patients receiving interventional cardiology are recorded in NAPCI.

Use of routine data has the potential to reduce the costs of conducting trials. The cost of the TASTE trial was reported as US$300,000, or approximately $50 per patient,[4] 2 per cent of the cost of a traditional randomised trial, but differs from the PARAMEDIC trial in that we did not use registry data to identify and recruit patients in the challenging and time-pressured setting of our-of-hospital cardiac arrest. In the West of Scotland Coronary Prevention (WOSCOPS) trial, data linkage reduced

For peer review only - http://bmjopen.bmj.com/site/about/guidelines.xhtml

costs of long term follow up to less than one per cent of trial budget.[23] However, the time cost of linkage could be unrealistic for some trials. Linkage for the PARAMEDIC trial took up to three years from application to the trial team obtaining the data. It has been suggested that NHS Digital, who performed the linkage to HES for our study was overwhelmed with data linkage applications.[24] This may limit the usefulness of administrative data in trials with funder-imposed deadlines for completion.

Limitations:

Our study had several limitations. The matching reliability was suboptimal due to relaxed matching criteria (using range of event date), matching methods and potential issues of data quality and completeness, common to administrative data. Bohensky et al.[25] conducted an evidence synthesis of data linkage studies and identified factors such as sub-optimal or incomplete linkage leading to systematic bias. They considered the participant or population characteristics that can influence the validity and completeness of data linkage and may in turn lead to systematic bias in reporting. They reported variation in quality of data linkage across geographical/hospital sites, which could be due to high staff turnover or not sufficient resources allocated to the data collection and/or coding. We have not considered such variations in this study, but overall match quality was high in the matched cases.

Secondly, routine data were not fully available for all patients transported to hospital. Some patients were not included in the linkage as their data were not available in HES at the time of our data application. Although no substantial bias was shown, the generalisability of results could be limited. Several data fields were incomplete, for example, MINAP captures most ST elevation myocardial infarction (STEMI) cases but data for non-STEMI are less complete. We also cannot confirm how many patients required specialist care and should be included in non-HES datasets. Therefore, we were unable to assess and report the impact of unmatched cases in in the linkage to these registries.

Thirdly, we used the first matched admission without considering repeated or later admissions. We were therefore unlikely to fully describe patients' hospital pathway based on matched information.

Fourthly, our focus for the present study was on assessing the feasibility of using administrative data for purposes of follow-up. We did not assess the utility of administrative data to facilitate recruitment of trial patients since this was considered unrealistic in the clinical context of cardiac arrest.

Fifthly, we did not assess the financial cost of manual data collection at hospitals to compare with the cost of the use of registries in the trial linkage.

For peer review only - http://bmjopen.bmj.com/site/about/guidelines.xhtml

Recommendations

Based on our experience, we made the following recommendations to improve the use of data linkage in trials:

1. When planning a trial using linkage to administrative registries, careful planning is required to assess availability of the required data. Linkage to routine data in different jurisdictions or multiple registries requires separate applications for data release and may be subject to data availability.
2. Trialists need to be mindful of prolonged processes for regulatory approvals, data release and validation. These processes may extend beyond trial funding.
3. Data linkage is a lengthy often unpredictable process in the application stage, possibly due to the restricted capacity of registries funded primarily to assess quality of care. Most registries in the NHS are funded as national audits and do not have sufficient resources for the timely processing of data sharing requests.
4. The quality of routinely collected data in the national registries may be inferior to that collected using traditional trial processes. Registry data are collected in high volume with limited resources and the validation process is unlikely to be as robust as in trials that are better resourced. Moreover, collected variables in registries are reviewed periodically and may change to reflect advances in clinical practice, which can impact on data completeness. Therefore, we suggest that trialists use registry data as the main source of all in-hospital data points and active data collection by a study team as an auxiliary approach to collect data for the unmatched patients.
5. It is common for registries to charge a fee for data release, which should be costed in to trial budgets.

Conclusions

This study shows that it is feasible to track patients from the pre-hospital setting through to hospital admission using routinely available administrative datasets with a moderate to high degree of success. This may improve the efficiency and reduce the costs for longer-term follow-up in cardiac arrest trials.

For peer review only

Declaration of interests

GDP, RL, TQ, CDD and SG report grants from NIHR HTA Programme during the conduct of the study.

The other authors declare no competing interests.

Author's contributions

CS, JH, SG and GDP led on the data linkage. CJ analysed data. CJ, TQ and LG drafted the manuscript with input from the co-authors; RL, CS, JH, CDD, HP, MAS, NR, SJM, SG and GDP reviewed the manuscript. All authors approved the final version.

Acknowledgments

This is a summary of independent research partly funded by the National Institute for Health Research's (NIHR) Health Technology Assessment Programme (Grant Reference Number HTA-07/37/69). The views expressed are those of the author(s) and not necessarily those of the NHS, the NIHR, of the Department of Health. GDP received support as a NIHR Senior Investigator. MS is supported as an NIHR Doctoral Research Fellow. We thank the independent members of the Trial Steering Committee (Jon Nicholl, Helen Snooks, Fionna Moore, Alasdair Gray, Martyn Box, Father Neil Bayliss (PPR), and John Long (PPR)) and the Data Monitoring Committee (Marion Campbell, Jerry Nolan, and Kathy Rowan). We acknowledge support from the OHCAO registry project that is funded by the British Heart Foundation and Resuscitation Council (UK).

Data sharing statement

No additional data sharing available.

For peer review only

References

1. Neumar RW, Shuster M, Callaway CW, et al. Part 1: Executive Summary: 2015 American Heart Association Guidelines Update for Cardiopulmonary Resuscitation and Emergency Cardiovascular Care. Circulation 2015;**132**(18 Suppl 2):S315-67. doi: 10.1161/CIR.0000000000000252
2. Whitehead L, Perkins GD, Clarey A, et al. A systematic review of the outcomes reported in cardiac arrest clinical trials: the need for a core outcome set. Resuscitation 2015;**88**:150-7. doi: 10.1016/j.resuscitation.2014.11.013
3. Lauer MS, Bonds D. Eliminating the "expensive" adjective for clinical trials. Am Heart J 2014;**167**(4):419-20. doi: 10.1016/j.ahj.2013.12.003
4. Frobert O, Lagerqvist B, Olivecrona GK, et al. Thrombus aspiration during ST-segment elevation myocardial infarction. N Engl J Med 2013;**369**(17):1587-97. doi: 10.1056/NEJMoa1308789
5. James S, Rao SV, Granger CB. Registry-based randomized clinical trials--a new clinical trial paradigm. Nat Rev Cardiol 2015;**12**(5):312-6. doi: 10.1038/nrcardio.2015.33
6. Lauer MS, D'Agostino RB, S.r. The randomized registry trial--the next disruptive technology in clinical research? N Engl J Med 2013;**369**(17):1579-81. doi: 10.1056/NEJMp1310102
7. Rao SV, Hess CN, Barham B, et al. A registry-based randomized trial comparing radial and femoral approaches in women undergoing percutaneous coronary intervention: the SAFE-PCI for Women (Study of Access Site for Enhancement of PCI for Women) trial. JACC Cardiovasc Interv 2014;**7**(8):857-67. doi: 10.1016/j.jcin.2014.04.007
8. Hofmann R, James SK, Svensson L, et al. DETermination of the role of OXygen in suspected Acute Myocardial Infarction trial. Am Heart J 2014;**167**(3):322-8. doi: 10.1016/j.ahj.2013.09.022
9. Perkins GD, Woollard M, Cooke MW, et al. Prehospital randomised assessment of a mechanical compression device in cardiac arrest (PaRAMeDIC) trial protocol. Scand J Trauma Resusc Emerg Med 2010;**18**:58. doi: 10.1186/1757-7241-18-58
10. Perkins GD, Lall R, Quinn T, et al. Mechanical versus manual chest compression for out-of-hospital cardiac arrest (PARAMEDIC): a pragmatic, cluster randomised controlled trial. Lancet 2015;**385**(9972):947-55. doi: 10.1016/S0140-6736(14)61886-9
11. Ji C, Lall R, Quinn T, et al. Post-admission outcomes of participants in the PARAMEDIC trial: A cluster randomised trial of mechanical or manual chest compressions. Resuscitation 2017;**118**:82-88. doi: 10.1016/j.resuscitation.2017.06.026
12. Marti J, Hulme C, Ferreira Z, et al. The cost-effectiveness of a mechanical compression device in out-of-hospital cardiac arrest. Resuscitation 2017;**117**:1-7. doi: 10.1016/j.resuscitation.2017.04.036
13. Rajagopal S, Kaye CR, Lall R, et al. Characteristics of patients who are not resuscitated in out of hospital cardiac arrests and opportunities to improve community response to cardiac arrest. Resuscitation 2016;**109**:110-15. doi: 10.1016/j.resuscitation.2016.09.014
14. Herrett E, Smeeth L, Walker L, et al. The Myocardial Ischaemia National Audit Project (MINAP). Heart 2010;**96**(16):1264-7. doi: 10.1136/hrt.2009.192328
15. Ludman PF, de Belder MA, McLenachan JM, et al. The importance of audit to monitor applications of procedures and improve primary angioplasty results. EuroIntervention 2012;**8 Suppl P**:P62-70. doi: 10.4244/EIJV8SPA11
16. Harrison DA, Brady AR, Rowan K. Case mix, outcome and length of stay for admissions to adult, general critical care units in England, Wales and Northern Ireland: the Intensive Care National Audit & Research Centre Case Mix Programme Database. Crit Care 2004;**8**(2):R99-111. doi: 10.1186/cc2834
17. Boyle MJ. The experience of linking Victorian emergency medical service trauma data. BMC Med Inform Decis Mak 2008;**8**:52. doi: 10.1186/1472-6947-8-52

For peer review only - http://bmjopen.bmj.com/site/about/guidelines.xhtml

18. Seymour CW, Kahn JM, Martin-Gill C, et al. Creating an infrastructure for comparative effectiveness research in emergency medical services. Acad Emerg Med 2014;**21**(5):599-607. doi: 10.1111/acem.12370
19. Mears GD, Rosamond WD, Lohmeier C, et al. A link to improve stroke patient care: a successful linkage between a statewide emergency medical services data system and a stroke registry. Acad Emerg Med 2010;**17**(12):1398-404. doi: 10.1111/j.1553-2712.2010.00925.x
20. Hettinger AZ, Cushman JT, Shah MN, et al. Emergency medical dispatch codes association with emergency department outcomes. Prehosp Emerg Care 2013;**17**(1):29-37. doi: 10.3109/10903127.2012.710716
21. Barry SJ, Dinnett E, Kean S, et al. Are routinely collected NHS administrative records suitable for endpoint identification in clinical trials? Evidence from the West of Scotland Coronary Prevention Study. PLoS One 2013;**8**(9):e75379. doi: 10.1371/journal.pone.0075379
22. Li G, Sajobi TT, Menon BK, et al. Registry-based randomized controlled trials- what are the advantages, challenges, and areas for future research? J Clin Epidemiol 2016;**80**:16-24. doi: 10.1016/j.jclinepi.2016.08.003
23. McCowan C. Using routinely collected clinical data to support clinical trials: a view from Scotland. Proceedings from the Clinical Trials Ontario 2015 Clinical Trials Conference 04-05 March 2015. http://www.ctontario.ca/cms/media/cto-2015-conference-speaker-presentation_colin-mccowan.pdf (accessed 29 November 2017).
24. Filippon J. Slow and costly access to anonymised patient data impedes academic research. BMJ 2015;**351**:h5087. doi: 10.1136/bmj.h5087

25. Bohensky MA, Jolley D, Sundararajan V, et al. Data linkage: a powerful research tool with potential problems. BMC Health Serv Res 2010;**10**:346. doi: 10.1186/1472-6963-10-346

For peer review only

For peer review only - http://bmjopen.bmj.com/site/about/guidelines.xhtml

For peer review only

For peer review only - http://bmjopen.bmj.com/site/about/guidelines.xhtml

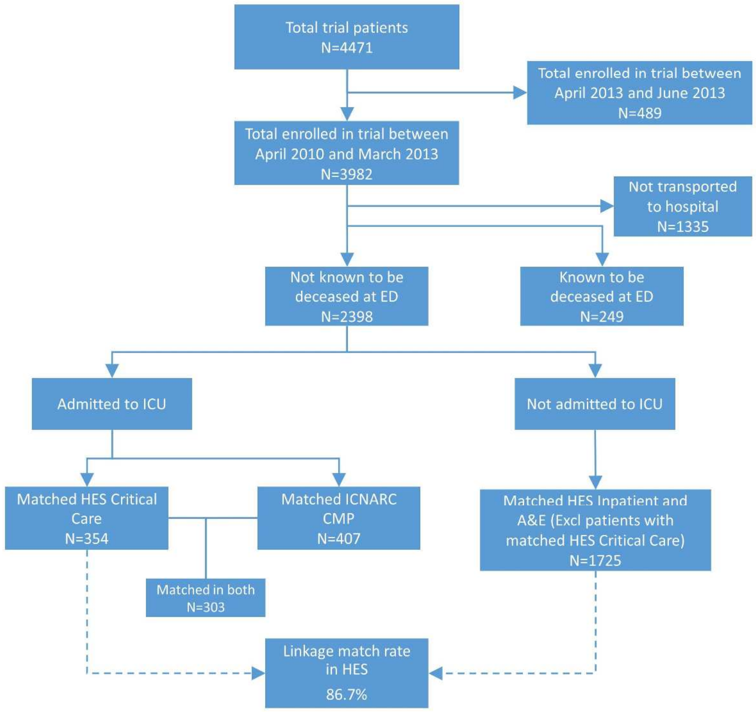

Figure 1: Linkage match rate and flowchart of patients retrieving HES or CMP data.

Legend: ICU, intensive care unit; ICNARC, Intensive Care National Audit and Research Centre; CMP, Case Mix Programme, HES, Hospital Episode Statistics; ED, Emergency Department; A&E, Accident and Emergency.

254x190mm (300 x 300 DPI)

Supplementary materials:

Supplementary Table 1: Summary of retrieved information in each linked dataset.

HES Inpatient		All
CSK operation	No	606 (79.6%)
	Yes	155 (20.4%)
Destination of discharge	The usual place of residence, including no fixed abode	192 (24.9%)
	Temporary place of residence when usually resident elsewhere	4 (0.5%)
	Repatriation from high security psychiatric hospital (from 1999-2000)	0 (0.0%)
	Repatriation from high security psychiatric hospital (from 1999-2000)	0 (0.0%)
	Penal establishment - court (from 1999-2000)	0 (0.0%)
	Penal establishment - police station (from 1999-2000)	0 (0.0%)
	Penal establishment - court and police station excluded (from 1999-2000 to 2006-07)	0 (0.0%)
	High security psychiatric hospital, Scotland (from 1999-2000)	0 (0.0%)
	NHS other hospital provider - high security psychiatric accommodation	0 (0.0%)
	NHS other hospital provider - medium secure unit (from 1999-2000)	32 (4.2%)
	NHS other hospital provider - ward for maternity patients or neonates	0 (0.0%)
	NHS other hospital provider - ward for patients who are mentally ill or have learning disabilities	0 (0.0%)
	NHS run nursing home, residential care home or group home	3 (0.4%)
	Local authority Part 3 residential accommodation - where care is provided (from 1996-97)	0 (0.0%)
	Local authority foster care, but not in Part 3 residential accommodation - where care is provided (from 1996-97)	0 (0.0%)
	LA home or care (1989-90 to 1995-96)	0 (0.0%)
	Non-NHS run hospital - medium secure unit (from 2003-04)	1 (0.1%)
	Non-NHS (other than local authority) run residential care home (from 1996-97 to 2003-04) and care home (from 2003-04)	3 (0.4%)
	Non-NHS (other than local authority) run nursing home (from 1996-97 to 2003-04)	0 (0.0%)
	Non-NHS run hospital	0 (0.0%)
	Non-NHS (other than local authority) run hospice	2 (0.3%)
	Non-NHS institution (1989-90 to 1995-96)	0 (0.0%)
	Not applicable	7 (0.9%)
	Not known	1 (0.1%)
	Died	522 (67.7%)
Method of discharge	Discharged on clinical advice or with clinical consent	234 (30.4%)
	Self discharged, or discharged by a relative or advocate	5 (0.6%)

For peer review only - http://bmjopen.bmj.com/site/about/guidelines.xhtml

	Discharged by a mental health review tribunal, the Home Secretary or a court	0 (0.0%)
	Baby was still born	0 (0.0%)
	Not applicable: patient still in hospital	10 (1.3%)
	Not known: a validation error	0 (0.0%)
	Died	522 (67.7%)
IMD 2004 index	Missing	84 (10.9%)
	Least deprived 10%	64 (8.3%)
	Less deprived 10-20%	43 (5.6%)
	Less deprived 20-30%	47 (6.1%)
	Less deprived 30-40%	35 (4.5%)
	Less deprived 40-50%	50 (6.5%)
	More deprived 10-20%	80 (10.4%)
	More deprived 20-30%	84 (10.9%)
	More deprived 30-40%	58 (7.5%)
	More deprived 40-50%	61 (7.9%)
	Most deprived 10%	165 (21.4%)
Hospital length of stay*		7.7 (20.3)
	**HES A&E**	**All**
A&E department type - A&E only	Emergency departments	1865 (96.8%)
	Consultant-led mono specialty accident and emergency service'	0 (0.0%)
	Other type of A&E. Excludes NHS walk-in centres	4 (0.2%)
	NHS walk-in centres	1 (0.1%)
	Not known	57 (3.0%)
A&E attendance disposal - A&E only	Admitted to hospital bed / became a lodged patient of the same health care provider	560 (29.1%)
	Discharged – follow-up treatment to be provided by general practitioner	5 (0.3%)
	Discharged – did not require any follow-up treatment	9 (0.5%)
	Referred to A&E clinic	13 (0.7%)
	Referred to fracture clinic	1 (0.1%)
	Referred to other outpatient clinic	1 (0.1%)
	Transferred to other healthcare provider	28 (1.5%)
	Referred to other healthcare professional	6 (0.3%)
	Left department before being treated	1 (0.1%)
	Left department having refused treatment	0 (0.0%)
	Other	5 (0.3%)
	Not known	2 (0.1%)
	Died in department	1296 (67.3%)
IMD 2004 index	Missing	7 (0.4%)
	Least deprived 10%	126 (6.5%)
	Less deprived 10-20%	95 (4.9%)
	Less deprived 20-30%	139 (7.2%)
	Less deprived 30-40%	120 (6.2%)

For peer review only - http://bmjopen.bmj.com/site/about/guidelines.xhtml

	Less deprived 40-50%	133 (6.9%)
	More deprived 10-20%	257 (13.3%)
	More deprived 20-30%	213 (11.1%)
	More deprived 30-40%	173 (9.0%)
	More deprived 40-50%	205 (10.6%)
	Most deprived 10%	459 (23.8%)
**HES Critical care**		**All**
Length of level 2 CC days		0.9 (1.7)
Length of level 3 CC days		4.4 (5.4)
Total length of CC days		6.6 (15.4)
**ICNARC CMP**		**All**
Length of level 0 ITU days		0 (0.3)
Length of level 1 ITU days		0.1 (0.6)
Length of level 2 ITU days		1.1 (4.0)
Length of level 3 ITU days		5.2 (9.4)
Total length of ITU days		6.5 (12.9)
Total length of ITU days in 30 days survived patients		10.5 (22.0)
Total length of ITU days in 30 days deceased patients		4.8 (4.5)
Days of alive and free of ITU stay in the first 28 days of cardiac arrest		6.9 (10.3)
Treatment withheld/withdrawn	Both withheld then withdrawn	23 (5.7%)
	Withheld	4 (1.0%)
	Withdrawn	133 (32.7%)
	Neither	247 (60.7%)
Organ donation	Heartbeating solid organ donor	13 (3.2%)
	No solid organs or tissues donated	199 (48.9%)
	Non-heartbeating solid organ donor	15 (3.7%)
	Tissue donor only	8 (2.0%)
**NICOR MINAP**		**All**
Admission Diagnosis	Definite myocardial infarction	126 (69.2%)
	Acute coronary syndrome	30 (16.5%)
	Chest pain cause	4 (2.2%)
	Other initial diagnosis	22 (12.1%)
Admission Ward	Missing	1 (0.5%)
	Cardiac care unit	94 (51.6%)
	Acute admissions unit	6 (3.3%)
	General medical ward	1 (0.5%)
	Intensive therapy unit	69 (37.9%)
	Other	9 (4.9%)
	Cardiac ward (non CCU)	1 (0.5%)
	Stepdown ward	0 (0.0%)
	Unknown	0 (0.0%)
	Died in A&E	1 (0.5%)

For peer review only - http://bmjopen.bmj.com/site/about/guidelines.xhtml

Initial Reperfusion Treatment	Missing	3 (2.2%)
	None	18 (13.1%)
	Thrombolytic treatment	0 (0.0%)
	pPCI in house	116 (84.7%)
	Referred for consideration for pPCI elsewhere	0 (0.0%)*
	Unknown	0 (0.0%)
Procedure performed	Missing	30(21.9%)
	No angiogram	2 (1.5%)
	Angiogram but no PCI	9 (6.6%)
	Angiogram and PCI	96 (70.1%)
	Unknown	0 (0.0%)
Coronary angiography	Missing	1(2.9%)
	Protocol driven investigation performed in this hospital	12(35.3%)
	Symptom driven investigation performed in this hospital	7 (20.6%)
	Protocol driven investigation performed at another hospital	0 (0.0%)
	Symptom driven investigation performed at another hospital	0 (0.0%)
	Planned after discharge	0 (0.0%)
	Not applicable	9 (6.6%)
	Patient refused	1 (2.9%)
	Not performed	5 (14.7%)
	Unknown	0 (0.0%)
Coronary intervention	Missing	6 (17.6%)
	Percutaneous coronary intervention	9 (26.5**%)**
	CABG	0 (0.0%)
	PCI planned after discharge	0 (0.0%)
	CABG planned after discharge	1(2.9%)
	Not applicable	5 (14.7%)
	Patient refused	1 (2.9%)
	Not performed or arranged	11 (32.4%)
	Unknown	0 (0.0%)
Assessment at non-interventional centre	Missing	5 (2.7%)
	No contact with a non interventional hospital	126 (69.2%)
	Patient remains in ambulance	0 (0.0%)
	A&E	30 (16.5%)
	Acute assessment unit	2 (1.1%)
	CCU / cardiac facility	1 (0.5%)
	Self referral	0 (0.0%)
	Already in hospital	2 (1.1%)
	Other	0 (0.0%)
	Unknown	16 (8.8%)
Assessment at interventional centre	Missing	59 (32.4%)
	Assessed in A&E	68 (37.4%)
	Acute assessment unit	1 (0.5%)
	CCU / cardiac facility	26 (14.3%)

For peer review only - http://bmjopen.bmj.com/site/about/guidelines.xhtml

	Catheter laboratory	28 (15.4%)
	Already in hospital	0 (0.0%)
	Unknown	0 (0.0%)
**NICOR PCI**		**All**
Cardiopulmonary resuscitation (CPR)	Missing	29 (28.7%)
	No	72 (71.3%)
	Yes	0 (0.0%)
Coronary artery bypass grafting (CABG)	Missing	0 (0.0%)
	No	101 (100%)
	Yes	0 (0.0%)
Ventilation	Missing	101 (100%)*
	No	0 (0.0%)
	Yes	0 (0.0%)
Hypothermia	Missing	101 (100%)*
	No	0 (0.0%)
	Yes	0 (0.0%)
Percutaneous coronary intervention (PCI)	Missing	101 (100%)
	No	0 (0.0%)
	Yes	0 (0.0%)
	No	0 (0.0%)
	Yes	0 (0.0%)

Note: *: Variables were added to the dataset after the linkage was performed.

For peer review only

For peer review only - http://bmjopen.bmj.com/site/about/guidelines.xhtml

Supplementary Table 2: Summary of demographic and cardiac arrest event characteristics in trial patients with and without matched HES inpatient, Critical care and A&E data

		HES matched N=2079	HES unmatched N=319	p value
Age (year)		73.6 (21.9)	71.8 (24.4)	0.151
Sex	Male	1315 (63.3%)	215 (67.4%)	0.151
	Female	764 (36.8%)	104 (32.6%)	
Initial aetiology	Presumed cardiac	1786 (85.9%)	272 (85.3%)	0.864
	Respiratory	146 (7.0%)	27 (8.5%)	
	Submersion	8 (0.4%)	1 (0.3%)	
	Other	74 (3.6%)	9 (2.8%)	
	Unknown	65 (3.1%)	10 (3.1%)	
Initial rhythm	VF	624 (30.0%)	99 (31.0%)	0.468
	VT	20 (1.0%)	1 (0.3%)	
	PEA	613 (29.5%)	83 (26.0%)	
	Asystole	727 (35.0%)	118 (37.0%)	
	Unknown	95 (4.6%)	18 (5.6%)	
Location	Home	1610 (77.4%)	206 (64.6%)	<0.001
	Public place	333 (16.0%)	89 (27.9%)	
	Other	136 (6.5%)	24 (7.5%)	
Witness	Not witnessed	508 (24.5%)	60 (18.8%)	0.027
	By bystander	961 (46.3%)	170 (53.3%)	
	By EMS	413 (19.9%)	60 (18.8%)	
	By Non-EMS healthcare	64 (3.1%)	4 (1.3%)	
	Unknown	132 (6.4%)	25 (7.8%)	
Cardiopulmonary resuscitation (CPR) by Bystander	No	1104 (53.1%)	156 (48.9%)	0.376
	Yes	862 (41.5%)	144 (45.1%)	
	Unknown	113 (5.4%)	19 (6.0%)	
Response time (minute)†		6.1 (4.3)	7.2 (5.1)	<.0001
Survival at 30days	Alive	224 (10.8%)	38 (11.9%)	0.544
	Deceased	1855 (89.2%)	281 (88.1%)	
ROSC at hospital transfer	ROSC	790 (38.0%)	125 (39.2%)	<0.001
	CPR in progress	1204 (57.9%)	135 (42.3%)	
	Unknown	85 (4.1%)	59 (18.5%)	
EQ5d at 3 months		70.0 (20.0)	75.0 (20.0)	0.970
EQ5d at 12 months		80.0 (30.0)	72.5 (26.0)	0.368
SF12 mental health at 3 months		50.4 (16.8)	45.4 (23.0)	0.690
SF12 physical health at 3 months		40.8 (14.2)	44.1 (15.0)	0.533
SF12 mental health at 12 months		51.0 (14.5)	44.1 (10.5)	0.118
SF12 physical health at 12 months		45.2 (17.4)	38.4 (13.9)	0.059
HADS Anxiety at 12 months		5.0 (7.0)	6.5 (5.0)	0.112

For peer review only - http://bmjopen.bmj.com/site/about/guidelines.xhtml

HADS depression at 12 months	4.0 (5.0)	6.0 (4.5)	0.163
MMSE at 12 months	29.0 (3.0)	29.0 (2.0)	0.648
PTSD at 12 months	27.0 (17.0)	33.0 (19.0)	0.280

Note: Continuous variables were shown as median (Interquartile range) and categorical variables were shown as n (percentage). †: response time was from 999 call to EMS arrival at scene.

For peer review only

For peer review only - http://bmjopen.bmj.com/site/about/guidelines.xhtml

Supplementary Table 3: Summary of demographic and cardiac arrest event characteristics in patients with matched HES Critical care only, CMP only and both.

		HES Critical Care only	ICNARC CMP only	Critical care & CMP	p value
Age (year)		68.3 (24.6)	64.8 (28.2)	65.5 (21.2)	0.684
Sex	Male	36 (70.6%)	66 (63.5%)	208 (68.7%)	0.556
	Female	15 (29.4%)	38 (36.5%)	95 (31.4%)	
Aetiology	Presumed cardiac	45 (88.2%)	84 (80.8%)	247 (81.5%)	0.554
	Respiratory	1 (2.0%)	12 (11.5%)	25 (8.3%)	
	Submersion	0 (0.0%)	1 (1.0%)	1 (0.3%)	
	Other	3 (5.9%)	6 (5.8%)	20 (6.6%)	
	Unknown	2 (3.9%)	1 (1.0%)	10 (3.3%)	
Location	Home	37 (72.6%)	68 (65.4%)	209 (69.0%)	0.516
	Public place	14 (27.5%)	30 (28.9%)	79 (26.1%)	
	Other	0 (0.0%)	6 (5.8%)	15 (5.0%)	
Witness	No	11 (21.6%)	26 (25%)	69 (22.8%)	0.981
	By bystander	29 (56.9%)	59 (56.7%)	174 (57.4%)	
	By EMS	7 (13.7%)	13 (12.5%)	32 (10.6%)	
	By Non-EMS healthcare	1 (2.0%)	2 (1.9%)	9 (3.0%)	
	Unknown	3 (5.9%)	4 (3.9%)	19 (6.3%)	
Cardiopulmonary resuscitation	No	24 (47.1%)	45 (43.3%)	136 (44.9%)	0.802
	Yes	24 (47.1%)	52 (50.0%)	155 (51.2%)	
	Unknown	3 (5.9%)	7 (6.7%)	12 (4.0%)	
Rhythm	VF	26 (51%)	44 (42.3%)	153 (50.5%)	0.437
	VT	2 (3.9%)	1 (1.0%)	4 (1.3%)	
	PEA	10 (19.6%)	25 (24.0%)	55 (18.2%)	
	Asystole	11 (21.6%)	26 (25.0%)	80 (26.4%)	
	Unknown	2 (3.9%)	8 (7.7%)	11 (3.6%)	
Response time (minute)†		6.6 (4.4)	6.7 (4.7)	6.0 (3.9)	0.034
Survival at 30days	Alive	19 (37.3%)	31 (29.8%)	93 (30.7%)	0.606
	Deceased	32 (62.8%)	73 (70.2%)	210 (69.3%)	
ROSC at hospital transfer	ROSC	42 (82.4%)	79 (76%)	241 (79.5%)	0.294
	CPR in progress	7 (13.7%)	14 (13.5%)	47 (15.5%)	
	Unknown	2 (3.9%)	11 (10.6%)	15 (5.0%)	
EQ5d at 3 months		67.5 (24.0)	75.5 (16.0)	70.0 (25.0)	0.838
EQ5d at 12 months		80.0 (14.0)	74.0 (20.0)	80.0 (25.0)	0.298
SF12 mental health at 3 months		48.4 (11)	48.3 (19.3)	49.3 (16.5)	0.794
SF12 physical health at 3 months		38.9 (13.2)	39.2 (13)	41.1 (14.2)	0.836
SF12 mental health at 12 months		51.6 (8)	43 (14.3)	47.8 (15.2)	0.543
SF12 physical health at 12 months		42.9 (21)	38.8 (14.6)	46.7 (15.6)	0.235

For peer review only - http://bmjopen.bmj.com/site/about/guidelines.xhtml

HADS Anxiety at 12 months	5.0 (5.0)	9.5 (6.5)	6.0 (6.0)	0.078
HADS depression at 12 months	4.0 (3.0)	6.0 (5.5)	5.0 (8.0)	0.368
MMSE at 12 months	28.0 (3.0)	29.0 (3.0)	29.0 (3.0)	0.717
PTSD at 12 months	30.0 (8.5)	40.0 (23.0)	29.0 (15.0)	0.631

Note: Continuous variables were shown as median (Interquartile range) and categorical variables were shown as frequency (percentage). †: response time is from 999 call to EMS arrival at scene.

For peer review only

For peer review only - http://bmjopen.bmj.com/site/about/guidelines.xhtml

STROBE Statement—checklist of items that should be included in reports of observational studies

	Item No.	Recommendation	Page No.	Relevant text from manuscript
**Title and abstract**	1	(*a*) Indicate the study's design with a commonly used term in the title or the abstract	1	
		(*b*) Provide in the abstract an informative and balanced summary of what was done and what was found	3	
**Introduction**				
Background/rationale	2	Explain the scientific background and rationale for the investigation being reported	5-6	
Objectives	3	State specific objectives, including any prespecified hypotheses	6	
**Methods**				
Study design	4	Present key elements of study design early in the paper	6	
Setting	5	Describe the setting, locations, and relevant dates, including periods of recruitment, exposure, follow-up, and data collection	6-7	
Participants	6	(*a*) *Cohort study*—Give the eligibility criteria, and the sources and methods of selection of participants. Describe methods of follow-up<br>*Case-control study*—Give the eligibility criteria, and the sources and methods of case ascertainment and control selection. Give the rationale for the choice of cases and controls<br>*Cross-sectional study*—Give the eligibility criteria, and the sources and methods of selection of participants	7	
		(*b*) *Cohort study*—For matched studies, give matching criteria and number of exposed and unexposed<br>*Case-control study*—For matched studies, give matching criteria and the number of controls per case	Not applicable	
Variables	7	Clearly define all outcomes, exposures, predictors, potential confounders, and effect modifiers. Give diagnostic criteria, if applicable	9	
Data sources/ measurement	8*	For each variable of interest, give sources of data and details of methods of assessment (measurement). Describe comparability of assessment methods if there is more than one group	6-7	
Bias	9	Describe any efforts to address potential sources of bias	8	
Study size	10	Explain how the study size was arrived at	6, 10 (figure 1)	

Continued on next page

For peer review only - http://bmjopen.bmj.com/site/about/guidelines.xhtml

Quantitative variables	11	Explain how quantitative variables were handled in the analyses. If applicable, describe which groupings were chosen and why	9
Statistical methods	12	(*a*) Describe all statistical methods, including those used to control for confounding	9
		(*b*) Describe any methods used to examine subgroups and interactions	Not applicable
		(*c*) Explain how missing data were addressed	Not applicable
		(*d*) *Cohort study*—If applicable, explain how loss to follow-up was addressed<br>*Case-control study*—If applicable, explain how matching of cases and controls was addressed<br>*Cross-sectional study*—If applicable, describe analytical methods taking account of sampling strategy	Not applicable
		(*e*) Describe any sensitivity analyses	Not applicable
**Results**			
Participants	13*	(a) Report numbers of individuals at each stage of study—eg numbers potentially eligible, examined for eligibility, confirmed eligible, included in the study, completing follow-up, and analysed	6-7
		(b) Give reasons for non-participation at each stage	Not applicable
		(c) Consider use of a flow diagram	10 (figure 1)
Descriptive data	14*	(a) Give characteristics of study participants (eg demographic, clinical, social) and information on exposures and potential confounders	Supplementary table 2
		(b) Indicate number of participants with missing data for each variable of interest	Supplementary table 1&2
		(c) *Cohort study*—Summarise follow-up time (eg, average and total amount)	Not applicable
Outcome data	15*	*Cohort study*—Report numbers of outcome events or summary measures over time	Not applicable
		Case-control study—Report numbers in each exposure category, or summary measures of exposure	Not applicable
		Cross-sectional study—Report numbers of outcome events or summary measures	10-11
Main results	16	(*a*) Give unadjusted estimates and, if applicable, confounder-adjusted estimates and their precision (eg, 95% confidence interval). Make clear which confounders were adjusted for and why they were included	10-11, Supplementary table 1-3
		(*b*) Report category boundaries when continuous variables were categorized	Not applicable
		(*c*) If relevant, consider translating estimates of relative risk into absolute risk for a meaningful time period	Not applicable

Continued on next page

For peer review only

For peer review only - http://bmjopen.bmj.com/site/about/guidelines.xhtml

Other analyses	17	Report other analyses done—eg analyses of subgroups and interactions, and sensitivity analyses	Not applicable
**Discussion**			
Key results	18	Summarise key results with reference to study objectives	11-12
Limitations	19	Discuss limitations of the study, taking into account sources of potential bias or imprecision. Discuss both direction and magnitude of any potential bias	4, 13
Interpretation	20	Give a cautious overall interpretation of results considering objectives, limitations, multiplicity of analyses, results from similar studies, and other relevant evidence	11-14
Generalisability	21	Discuss the generalisability (external validity) of the study results	13-14
**Other information**			
Funding	22	Give the source of funding and the role of the funders for the present study and, if applicable, for the original study on which the present article is based	15

*Give information separately for cases and controls in case-control studies and, if applicable, for exposed and unexposed groups in cohort and cross-sectional studies.

For peer review only

For peer review only - http://bmjopen.bmj.com/site/about/guidelines.xhtml
